# Nanopore sequencing-based genome assembly and evolutionary genomics of circum-basmati rice

**DOI:** 10.1186/s13059-020-1938-2

**Published:** 2020-02-05

**Authors:** Jae Young Choi, Zoe N. Lye, Simon C. Groen, Xiaoguang Dai, Priyesh Rughani, Sophie Zaaijer, Eoghan D. Harrington, Sissel Juul, Michael D. Purugganan

**Affiliations:** 1 Center for Genomics and Systems Biology, Department of Biology, New York University, New York, NY USA; 2 Oxford Nanopore Technologies, New York, NY USA; 3 New York Genome Center, New York, NY USA; 4 Center for Genomics and Systems Biology, NYU Abu Dhabi Research Institute, New York University Abu Dhabi, Abu Dhabi, United Arab Emirates

**Keywords:** *Oryza sativa*, Asian rice, Aromatic rice group, Domestication, Crop evolution, Nanopore sequencing, Aus, Basmati, Indica, Japonica, Admixture, Awnless, De novo genome assembly

## Abstract

**Background:**

The circum-basmati group of cultivated Asian rice (*Oryza sativa*) contains many iconic varieties and is widespread in the Indian subcontinent. Despite its economic and cultural importance, a high-quality reference genome is currently lacking, and the group’s evolutionary history is not fully resolved. To address these gaps, we use long-read nanopore sequencing and assemble the genomes of two circum-basmati rice varieties.

**Results:**

We generate two high-quality, chromosome-level reference genomes that represent the 12 chromosomes of *Oryza*. The assemblies show a contig N50 of 6.32 Mb and 10.53 Mb for Basmati 334 and Dom Sufid, respectively. Using our highly contiguous assemblies, we characterize structural variations segregating across circum-basmati genomes. We discover repeat expansions not observed in japonica—the rice group most closely related to circum-basmati—as well as the presence and absence variants of over 20 Mb, one of which is a circum-basmati-specific deletion of a gene regulating awn length. We further detect strong evidence of admixture between the circum-basmati and circum-aus groups. This gene flow has its greatest effect on chromosome 10, causing both structural variation and single-nucleotide polymorphism to deviate from genome-wide history. Lastly, population genomic analysis of 78 circum-basmati varieties shows three major geographically structured genetic groups: Bhutan/Nepal, India/Bangladesh/Myanmar, and Iran/Pakistan.

**Conclusion:**

The availability of high-quality reference genomes allows functional and evolutionary genomic analyses providing genome-wide evidence for gene flow between circum-aus and circum-basmati, describes the nature of circum-basmati structural variation, and reveals the presence/absence variation in this important and iconic rice variety group.

## Background

*Oryza sativa* or Asian rice is an agriculturally important crop that feeds one-half of the world’s population [1] and supplies 20% of people’s caloric intake (www.fao.org). Historically, *O. sativa* has been classified into two major variety groups, japonica and indica, based on morphometric differences and molecular markers [2, 3]. These variety groups can be considered as subspecies, particularly given the presence of reproductive barriers between them [4]. Archaeobotanical remains suggest japonica rice was domesticated ~ 9000 years ago in the Yangtze Basin of China, while indica rice originated ~ 4000 years ago when domestication alleles were introduced from japonica into either *O. nivara* or a proto-indica in the Indian subcontinent [5]. More recently, two additional variety groups have been recognized that are genetically distinct from japonica and indica: the aus/circum-aus and aromatic/circum-basmati rice [6–8].

The rich genetic diversity of Asian rice is likely a result from a complex domestication process involving multiple wild progenitor populations and the exchange of important domestication alleles between *O. sativa* variety groups through gene flow [5, 7, 9–17]. Moreover, many agricultural traits within rice are variety group-specific [18–23], suggesting local adaptation to environments or cultural preferences have partially driven the diversification of rice varieties.

Arguably, the circum-basmati rice group has been the least studied among the four major variety groups, and it was only recently defined in more detail based on insights from genomic data [7]. Among its members, the group boasts the iconic basmati rice (sensu stricto) from southern Asia and the sadri rice from Iran [6]. Many, but not all, circum-basmati varieties are characterized by distinct and highly desirable fragrance and texture [24]. Nearly all fragrant circum-basmati varieties possess a loss-of-function mutation in the *BADH2* gene that has its origins in ancestral japonica haplotypes, suggesting that an introgression between circum-basmati and japonica may have led to fragrant basmati rice [21, 25, 26]. Genome-wide polymorphism analysis of a smaller array of circum-basmati rice cultivars shows close association with japonica varieties [7, 16, 27], providing evidence that at least part of the genomic make-up of circum-basmati rice may indeed be traced back to japonica.

Whole-genome sequences are an important resource for evolutionary geneticists studying plant domestication, as well as breeders aiming to improve crop varieties. Single-molecule sequencing regularly produces sequencing reads in the range of kilobases (kb) [28]. This is particularly helpful for assembling plant genomes, which are often highly repetitive and heterozygous, and commonly underwent at least one round of polyploidization in the past [29–31]. The *Oryza sativa* genome, with a relatively modest size of ~ 400 Mb, was the first crop genome sequence assembled [29], and there has been much progress in generating de novo genome assemblies for other members of the genus *Oryza*. Currently, there are assemblies for nine wild species (*Leersia perrieri* [outgroup], *O. barthii*, *O. brachyantha*, *O. glumaepatula*, *O. longistaminata*, *O. meridionalis*, *O. nivara*, *O. punctata*, and *O. rufipogon*) and two domesticated species (*O. glaberrima* and *O. sativa*) [32–37].

Within domesticated Asian rice (*O. sativa*), genome assemblies are available for cultivars in most variety groups [32, 33, 38–42]. However, several of these reference assemblies are based on short-read sequencing data and show higher levels of incompleteness compared to assemblies generated from long-read sequences [40, 41]. Nevertheless, these de novo genome assemblies have been critical in revealing genomic variation (e.g., variations in genome structure and repetitive DNA, and de novo species- or population-specific genes) that were otherwise missed from analyzing a single reference genome. Recently, a genome assembly based on short-read sequencing data was generated for basmati rice [42]. Not only were there missing sequences in this assembly, it was also generated from DNA of an elite basmati breeding line. Such modern cultivars are not the best foundations for domestication-related analyses due to higher levels of introgression from other rice populations during modern breeding.

Here, we report the de novo sequencing and assembly of the landraces (traditional varieties) Basmati 334 [21, 43, 44] and Dom Sufid [21, 24, 45, 46] using the long-read nanopore sequencing platform of Oxford Nanopore Technologies [47]. Basmati 334 is from Pakistan, evolved in a rainfed lowland environment and is known to be drought tolerant at the seedling and reproductive stages [44]. It also possesses several broad-spectrum bacterial blight resistance alleles [48, 49], making Basmati 334 desirable for breeding resilience into modern basmati cultivars [49, 50]. Dom Sufid is an Iranian sadri cultivar that, like other sadri and basmati (sensu stricto) varieties, is among the most expensive varieties currently available in the market [24]. It has desirable characteristics such as aromaticity and grain elongation during cooking, although it is susceptible to disease and abiotic stress [24, 51]. Because of their special characteristics, both Basmati 334 and Dom Sufid are used in elite rice breeding programs to create high yielding and resilient aromatic rice varieties [24, 44–46, 50].

Based on long reads from nanopore sequencing, our genome assemblies have high quality, contiguity, and genic completeness, making them comparable in quality to assemblies associated with key rice reference genomes. We used our circum-basmati genome assemblies to characterize genomic variation existing within this important rice variety group, and analyze domestication-related and other evolutionary processes that shaped this variation. Our circum-basmati rice genome assemblies will be valuable complements to the available assemblies for other rice cultivars, unlocking important genomic variation for rice crop improvement.

## Results

### Nanopore sequencing of basmati and sadri rice

Using Oxford Nanopore Technologies’ long-read sequencing platform, we sequenced the genomes of the circum-basmati landraces Basmati 334 (basmati sensu stricto) and Dom Sufid (sadri). We called 1,372,950 reads constituting a total of 29.2 Gb for Basmati 334 and 1,183,159 reads constituting a total of 24.2 Gb for Dom Sufid (Table 1). For both samples, the median read length was > 17 kb, the read length N50 was > 33 kb, and the median quality score per read was ~ 11.

**Table 1:** Summary of nanopore sequencing read data

Flowcell	Number of reads	Median read length	Read length N50	Median quality score (QS)	Total bases
	Basmati 334				
FAK30515	288,473	19,905	36,743	11.27	6,843,069,570
FAK30732	306,247	18,792	30,974	11.19	6,341,851,953
FAK30522	228,191	17,366	36,456	11.07	4,816,938,523
FAK27872	244,606	18,335	31,267	11.35	5,045,781,146
FAK27919	305,433	18,087	30,727	11.43	6,191,306,294
All	1,372,950	18,576	33,005	11.27	29,238,947,486
	Dom Sufid				
FAK30464	300,290	18,477	37,754	11.34	6,681,819,859
FAK30582	258,584	17,641	34,213	11.30	5,353,774,444
FAK28890	330,924	16,756	34,033	10.96	6,553,200,184
FAK30064	293,361	16,178	32,835	10.99	5,618,557,776
All	1,183,159	17,237	34,728	11.14	24,207,352,263

### De novo assembly of the Basmati 334 and Dom Sufid rice genomes

Incorporating only those reads that had a mean quality score of > 8 and read lengths of > 8 kb, we used a total of 1,076,192 reads and 902,040 reads for the Basmati 334 and Dom Sufid genome assemblies, which resulted in a genome coverage of ~ 62× and ~ 51×, respectively (Table 2). We polished the genome assemblies with both nanopore and short Illumina sequencing reads. The final, polished genome assemblies spanned 386.5 Mb across 188 contigs for Basmati 334 and 383.6 Mb across 116 contigs for Dom Sufid. The genome assemblies had high contiguity, with a contig N50 of 6.32 Mb and 10.53 Mb for Basmati 334 and Dom Sufid, respectively. Our genome assemblies recovered more than 97% of the 1440 BUSCO [52] embryophyte gene groups, which is comparable to the BUSCO statistics for the japonica Nipponbare [33] (98.4%) and indica R498 reference genomes [41] (98.0%). This is an improvement from the currently available genome assembly of basmati variety GP295-1 [42], which was generated from Illumina short-read sequencing data and has a contig N50 of 44.4 kb with 50,786 assembled contigs.

**Table 2:** Summary of the circum-basmati rice genome assemblies

	Basmati 334	Dom Sufid
Genome coverage	62.5×	51.4×
Number of contigs	188	116
Total number of bases in contigs	386,555,741	383,636,250
Total number of bases scaffolded	386,050,525	383,245,802
Contig N50 length	6.32 Mb	10.53 Mb
Contig L50	20	13
Total contigs > 50 kbp	159	104
Maximum contig length	17.04 Mb	26.82 Mb
BUSCO gene completion (assembly)	97.6%	97.0%
GC content	43.6%	43.7%
Repeat content	44.4%	44.2%
Number of annotated genes	41,270	38,329
BUSCO gene completion (annotation)	95.4%	93.6%

We examined coding sequences of our circum-basmati genomes by conducting gene annotation using published rice gene models and the *MAKER* gene annotation pipeline [52, 53]. A total of 41,270 genes were annotated for the Basmati 334 genome, and 38,329 for the Dom Sufid genome. BUSCO gene completion analysis [52] indicated that 95.4% and 93.6% of the 3278 single-copy genes from the liliopsida gene dataset were found in the Basmati 334 and Dom Sufid gene annotations, respectively.

### Whole-genome comparison to other rice variety group genomes

**Fig. 1:**
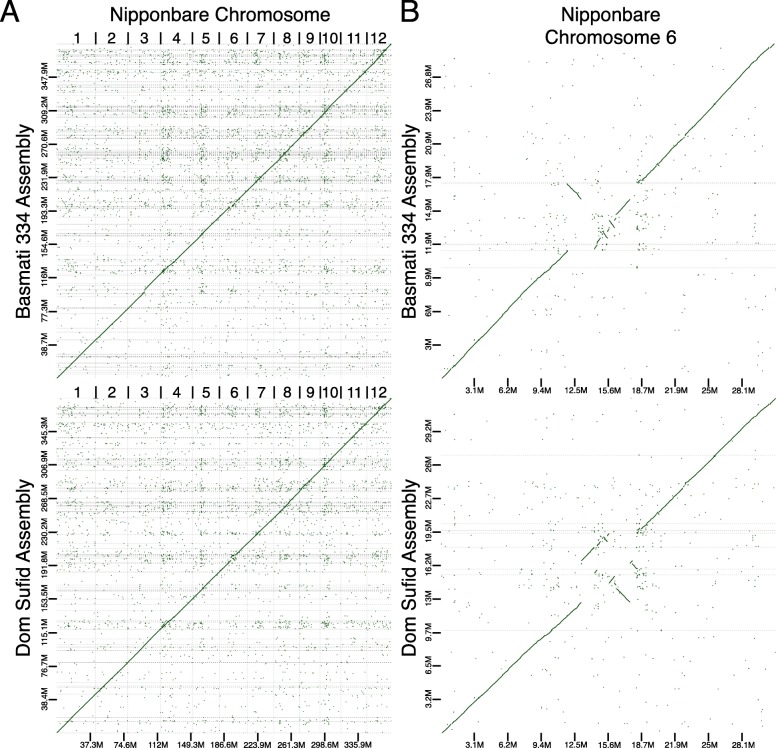
Dot plot comparing the assembly contigs of Basmati 334 and Dom Sufid to **a** all chromosomes of the Nipponbare genome assembly and **b** only chromosome 6 of Nipponbare. Only alignment blocks with greater than 80% sequence identity are shown

We aligned our draft genome assemblies to the japonica Nipponbare reference genome sequence [33], which represents one of the highest quality reference genome sequences (Fig. 1a). Between the Nipponbare, Basmati 334 and Dom Sufid genomes, high levels of macro-synteny were evident across the japonica chromosomes. Specifically, we observed little large-scale structural variation between Basmati 334 and Dom Sufid contigs and the japonica genome. A noticeable exception was an apparent inversion in the circum-basmati genome assemblies at chromosome 6 between positions 12.5 and 18.7 Mb (Nipponbare coordinates), corresponding to the pericentromeric region [54]. Interestingly, the same region showed an inversion between the Nipponbare and indica R498 reference genomes [41], whereas in the circum-aus N22 cultivar no inversions are observed (Additional file 1: Figure S1). While the entire region was inverted in R498, the inversion positions were disjoint in Basmati 334 and Dom Sufid, apparently occurring in multiple regions of the pericentromere. We independently verified the inversions by aligning raw nanopore sequencing reads to the Nipponbare reference genome using the long-read-aware aligner *ngmlr* [55], and the structural variation detection program *sniffles* [55]. *Sniffles* detected several inversions, including a large inversion between positions 13.1 and 17.7 Mb and between 18.18 and 18.23 Mb, with several smaller inversions located within the largest inversion (Additional file 2: Table S1).

Because of high macro-synteny with japonica (Fig. 1a), we ordered and oriented the contigs of the Basmati 334 and Dom Sufid assemblies using a reference genome-based scaffolding approach [56]. For both Basmati 334 and Dom Sufid, over 99.9% of the assembled genomic contigs were anchored to the Nipponbare reference genome (Table 2). The scaffolded circum-basmati chromosomes were similar in size to those in reference genomes for cultivars in other rice variety groups (Nipponbare [33], the circum-aus variety N22 [37], and the indica varieties IR8 [37] and R498 [41]) that were sequenced, assembled, and scaffolded to near completion (Table 3).

**Table 3:** Comparison of assembled chromosome sizes for cultivars across variety groups

Chromosome	Basmati 334	Dom Sufid	Nipponbare	N22	IR8	R498
1	44,411,451	44,306,286	43,270,923	44,711,178	44,746,683	44,361,539
2	35,924,761	36,365,206	35,937,250	38,372,633	37,475,564	37,764,328
3	40,305,655	38,133,813	36,413,819	36,762,248	39,065,119	39,691,490
4	34,905,232	34,714,597	35,502,694	33,558,078	35,713,470	35,849,732
5	30,669,872	31,017,353	29,958,434	28,792,057	31,269,760	31,237,231
6	29,982,228	32,412,977	31,248,787	29,772,976	32,072,649	32,465,040
7	30,410,531	29,511,326	29,697,621	29,936,233	30,380,234	30,277,827
8	29,921,941	29,962,976	28,443,022	25,527,801	30,236,384	29,952,003
9	24,050,083	23,970,096	23,012,720	22,277,206	24,243,884	24,760,661
10	25,596,481	24,989,786	23,207,287	20,972,683	25,246,678	25,582,588
11	29,979,012	29,949,236	29,021,106	29,032,419	32,337,678	31,778,392
12	29,893,278	27,912,150	27,531,856	22,563,585	25,963,606	26,601,357
Total	386,050,525	383,245,802	373,245,519	362,279,097	388,751,709	390,322,188

Next, we assessed the assembly quality of the circum-basmati genomes by contrasting them against available de novo-assembled genomes within the Asian rice complex (see the “Materials and methods” section for a complete list of genomes). We generated a multi-genome alignment to the Nipponbare genome, which we chose as the reference since its assembly and gene annotation is a product of years of community-based efforts [33, 57, 58]. To infer the quality of the gene regions in each of the genome assemblies, we used the multi-genome alignment to extract the coding DNA sequence of each Nipponbare gene and its orthologous regions from each non-japonica genome. The orthologous genes were counted for missing DNA sequences (“N” sequences) and gaps to estimate the percent of Nipponbare genes covered. For all genomes, the majority of Nipponbare genes had a near-zero proportion of sites that were missing in the orthologous non-Nipponbare genes (Additional file 1: Figure S2). The missing proportions of Nipponbare-orthologous genes within the Basmati 334 and Dom Sufid genomes were comparable to those for genomes that had higher assembly contiguity [37, 40, 41].

Focusing on the previously sequenced basmati GP295-1 genome [42], our newly assembled circum-basmati genomes had noticeably lower proportions of missing genes (Additional file 1: Figure S2). Furthermore, over 96% of base pairs across the Nipponbare genome were alignable against the Basmati 334 (total of 359,557,873 bp [96.33%] of Nipponbare genome) or Dom Sufid (total of 359,819,239 bp [96.40%] of Nipponbare genome) assemblies, while only 194,464,958 bp (52.1%) of the Nipponbare genome were alignable against the GP295-1 assembly.

We then counted the single-nucleotide and insertion/deletion (indel, up to ~ 60 bp) differences between the circum-basmati and Nipponbare assemblies to assess the overall quality of our newly assembled genomes. To avoid analyzing differences across unconstrained repeat regions, we specifically examined regions where there were 20 exact base-pair matches flanking a site that had a single-nucleotide or indel difference between the circum-basmati and Nipponbare genomes. In the GP295-1 genome, there were 334,500 (0.17%) single-nucleotide differences and 44,609 (0.023%) indels compared to the Nipponbare genome. Our newly assembled genomes had similar proportions of single-nucleotide differences with the Nipponbare genome, where the Basmati 334 genome had 780,735 (0.22%) differences and the Dom Sufid genome had 731,426 (0.20%). For indels, the Basmati 334 genome had comparable proportions of differences with 104,282 (0.029%) variants, but the Dom Sufid genome had higher proportions with 222,813 (0.062%) variants. In sum, our draft circum-basmati genomes had high contiguity and completeness as evidenced by assembly to the chromosome level and comparison to the Nipponbare genome. In addition, our genome assemblies were comparable to the Illumina sequence-generated GP295-1 genome for the proportion of genomic differences with the Nipponbare genome, suggesting they had high quality and accuracy as well.

Our circum-basmati genome assemblies should also be of sufficiently high quality for detailed gene-level analysis. For instance, a hallmark of many circum-basmati rice is aromaticity, and a previous study had determined that Dom Sufid, but not Basmati 334, is a fragrant variety [21]. We examined the two genomes to verify the presence or absence of the mutations associated with fragrance. There are multiple different loss-of-function mutations in the *BADH2* gene that cause rice varieties to be fragrant [21, 25, 26], but the majority of fragrant rice carry a deletion of 8 nucleotides at position chr8:20,382,861-20,382,868 of the Nipponbare genome assembly (version Os-Nipponbare-Reference-IRGSP-1.0). Using the genome alignment, we extracted the *BADH2* sequence region to compare the gene sequence of the non-fragrant Nipponbare to that of Basmati 334 and Dom Sufid. Consistent with previous observations [21], we found that the genome of the non-fragrant Basmati 334 did not carry the deletion and contained the wild-type *BADH2* haplotype observed in Nipponbare. The genome of the fragrant Dom Sufid, on the other hand, carried the 8-bp deletion, as well as the 3 single-nucleotide polymorphisms flanking the deletion. This illustrates that the Basmati 334 and Dom Sufid genomes are accurate enough for gene-level analysis.

### Circum-basmati gene analysis

**Fig. 2:**
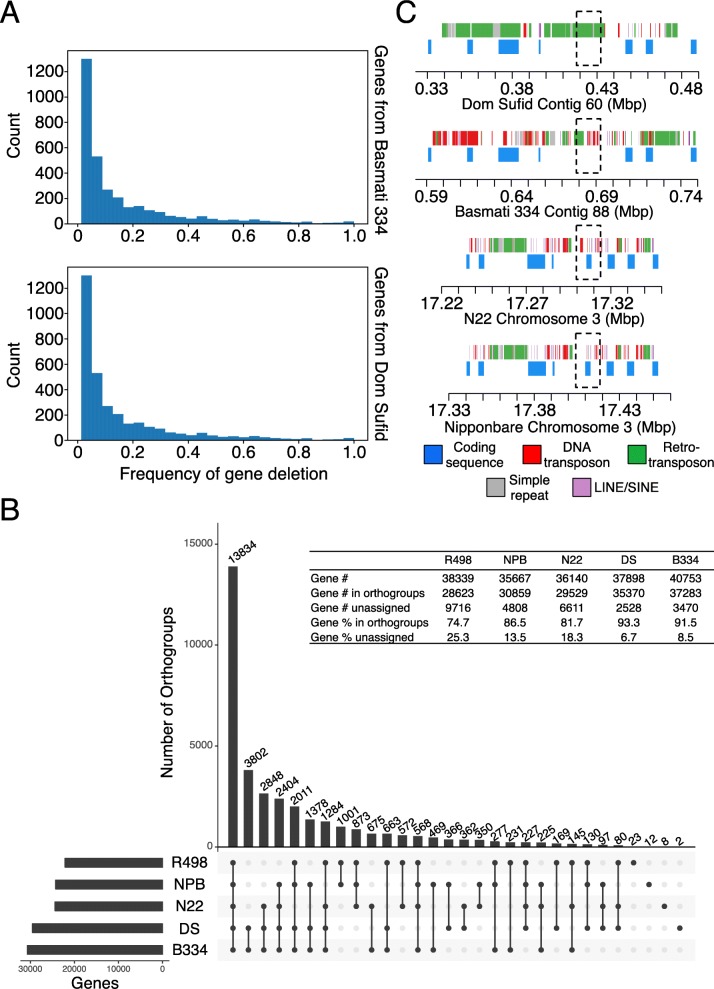
Circum-basmati gene sequence evolution. **a** The deletion frequency of genes annotated from the Basmati 334 and Dom Sufid genomes. Frequency was estimated from sequencing data on a population of 78 circum-basmati varieties. **b** Groups of orthologous and paralogous genes (i.e., orthogroups) identified in the reference genomes of circum-aus N22, japonica Nipponbare (NPB), and indica R498, as well as the circum-basmati genome assemblies Basmati 334 (B334) and Dom Sufid (DS) of this study. **c** Visualization of the genomic region orthologous to the Nipponbare gene Os03g0418600 (*Awn3-1*) in the N22, Basmati 334, and Dom Sufid genomes. Regions orthologous to *Awn3-1* are indicated with a dotted box

Our annotation identified ~ 40,000 coding sequences in the circum-basmati assemblies. We examined population frequencies of the annotated gene models across a circum-basmati population dataset to filter out mis-annotated gene models or genes at very low frequency in a population. We obtained Illumina sequencing reads from varieties included in the 3K Rice Genome Project [7] and sequenced additional varieties to analyze a total of 78 circum-basmati cultivars (see Additional file 2: Table S2 for a list of varieties). The Illumina sequencing reads were aligned to the circum-basmati genomes, and if the average coverage of a genic region was < 0.05× for an individual, this gene was called as a deletion in that variety. Since we used a low threshold for calling a deletion, the genome-wide sequencing coverage of a variety did not influence the number of gene deletions detected (Additional file 1: Figure S3). Results showed that gene deletions were indeed rare across the circum-basmati population (Fig. 2a), consistent with their probable deleterious nature. We found that 31,565 genes (76.5%) in Basmati 334 and 29,832 genes (77.8%) in the Dom Sufid genomes did not have a deletion across the population (see Additional file 2: Table S3 for a list of genes).

There were 517 gene models from Basmati 334 and 431 gene models from Dom Sufid that had a deletion frequency of ≥ 0.3 (see Additional file 2: Table S4 for a list of genes). These gene models with high deletion frequencies were not considered further in this analysis. The rest were compared against the circum-aus N22, indica R498, and japonica Nipponbare gene models to determine their orthogroup status (Fig. 2b; see Additional file 2: Table S5 for a list of genes and their orthogroup status), which are sets of genes that are orthologs and recent paralogs of each other [59].

The most frequent orthogroup class observed was for groups in which every rice variety group has at least one gene member. There were 13,894 orthogroups within this class, consisting of 17,361 genes from N22, 18,302 genes from Basmati 334, 17,936 genes from Dom Sufid, 17,553 genes from R498, and 18,351 genes from Nipponbare. This orthogroup class likely represents the set of core genes of *O. sativa* [42]. The second-highest orthogroup class observed was for groups with genes that were uniquely found in both circum-basmati genomes (3802 orthogroups). These genes represent those restricted to the circum-basmati group.

In comparison to genes in other rice variety groups, the circum-basmati genes shared the highest number of orthogroups with circum-aus (2648 orthogroups), followed by japonica (1378 orthogroups), while sharing the lowest number of orthogroups with indica (663 orthogroups). In fact, genes from indica variety R498 had the lowest number assigned to an orthogroup (Fig. 2b inset table), suggesting this genome had more unique genes, i.e., without orthologs/paralogs to genes in other rice variety groups.

### Genome-wide presence/absence variation within the circum-basmati genomes

Our assembled circum-basmati genomes were > 10 Mb longer than the Nipponbare genome, but individual chromosomes showed different relative lengths (Table 3) suggesting a considerable number of presence/absence variants (PAVs) between the genomes. We examined the PAVs between the circum-basmati and Nipponbare genomes using two different computational packages: (i) *sniffles*, which uses raw nanopore reads aligned to a reference genome to call PAVs, and (ii) *assemblytics* [60], which aligns the genome assemblies to each other and calls PAVs. The results showed that, while the total number of PAVs called by *sniffles* and *assemblytics* were similar, only ~ 36% of PAVs had overlapping positions (Table 4). In addition, the combined total size of PAVs was larger for predictions made by *sniffles* compared to those by *assemblytics*. For subsequent analysis, we focused on PAVs that were called by both methods.

**Table 4:** Comparison of presence/absence variation called by two different computational packages

	*Sniffles*	*Assemblytics*	Overlap
Basmati 334			
Deletion counts	11,989	11,247	4051
Deleted base-pairs	43,768,763	29,048,238	19,328,854
Insertion counts	11,447	12,161	3734
Inserted base-pairs	19,650,518	14,498,550	5,783,551
Dom Sufid			
Deletion counts	9901	10,115	3649
Deleted base-pairs	36,600,114	26,128,143	17,274,967
Insertion counts	9834	11,134	3340
Inserted base-pairs	16,527,995	12,902,410	5,160,503

**Fig. 3:**
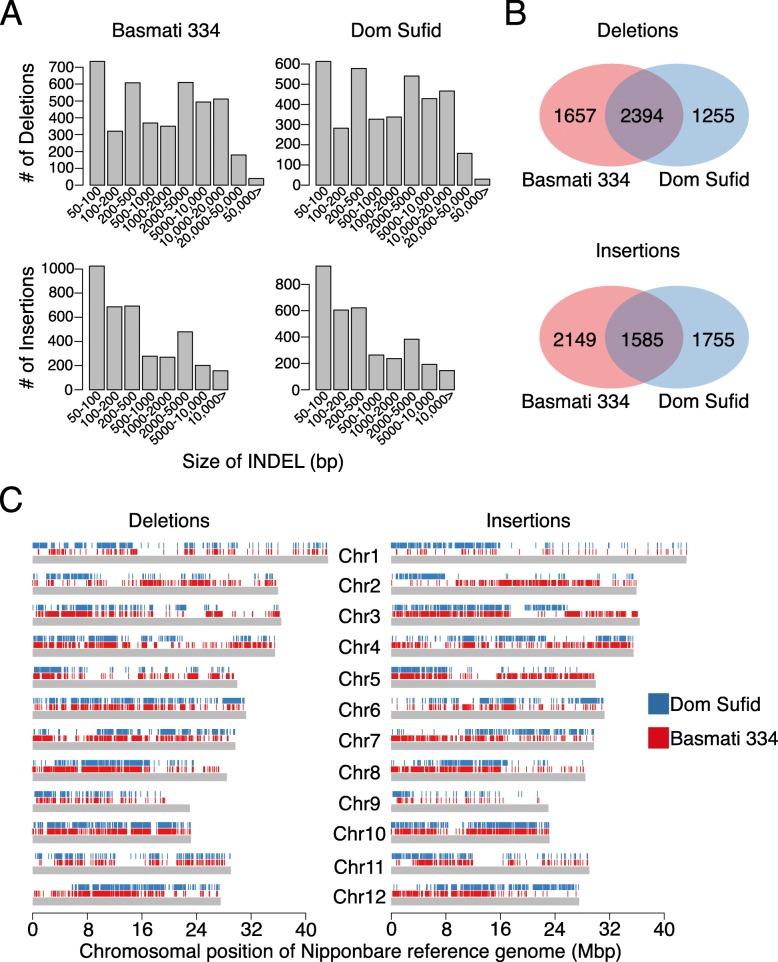
Presence/absence variation across the circum-basmati rice genome assemblies. **a** Distribution of the presence/absence variant sizes compared to the japonica Nipponbare reference genome. **b** Number of presence/absence variants that are shared between or unique for the circum-basmati genomes. **c** Chromosome-wide distribution of presence/absence variation for each circum-basmati rice genome, relative to the Nipponbare genome coordinates

The distribution of PAV sizes indicated that large PAVs were rare across the circum-basmati genomes, while PAVs < 500 bps in size were the most common (Fig. 3a). Within smaller-sized PAVs those in the 200–500-bp size range showed a peak in abundance. A closer examination revealed that sequence positions of more than 75% of these 200–500-bp-sized PAVs overlapped with transposable element coordinates in the circum-basmati genomes (Additional file 2: Table S6). A previous study based on short-read Illumina sequencing data reported a similar enrichment of short repetitive elements such as the long terminal repeats (LTRs) of retrotransposons, *Tc1/mariner* elements, and *mPing* elements among PAVs in this size range [61].

PAVs shorter than 200 bps also overlapped with repetitive sequence positions in the circum-basmati genomes, but the relative abundance of each repeat type differed among insertion and deletion variants. Insertions in the Basmati 334 and Dom Sufid genomes had a higher relative abundance of simple sequence repeats (i.e., microsatellites) compared to deletions (Additional file 2: Table S6). These inserted simple sequence repeats were highly enriched for (AT)_n_ dinucleotide repeats, which in Basmati 334 accounted for 66,624 bps out of a total of 72,436 bps (92.0%) of simple sequence repeats, and for Dom Sufid 56,032 bps out of a total of 63,127 bps (88.8%).

Between the Basmati 334 and Dom Sufid genomes, ~ 45% of PAVs had overlapping genome coordinates (Fig. 3b) suggesting that variety-specific insertion and deletion polymorphisms were common. We plotted PAVs for each of our circum-basmati genomes to visualize their distribution (Fig. 3c). Chromosome-specific differences in the distribution of PAVs were seen for each circum-basmati genome: in Basmati 334, for example, chromosome 1 had the lowest density of PAVs, while in Dom Sufid this was the case for chromosome 2 (Additional file 1: Figure S4). On the other hand, both genomes showed significantly higher densities of PAVs on chromosome 10 (Tukey’s range test *p* < 0.05). This suggested that, compared to Nipponbare, chromosome 10 was the most differentiated in terms of insertion and deletion variations in both of our circum-basmati genomes.

### Evolution of circum-basmati rice group-specific gene presence and absence variation

The proportion of repeat sequences found within the larger-sized PAVs (i.e., those > 2 kb) was high, where between 84 and 98% of large PAVs contained transposable element-related sequences (Additional file 2: Table S6). Regardless, these larger PAVs also involved loss or gain of coding sequences. For instance, gene ontology analysis of domesticated rice gene orthogroups showed enrichment for genes related to electron transporter activity among both circum-basmati-specific gene losses and gains (see Additional file 2: Table S7 for gene ontology results for circum-basmati-specific gene losses and Additional file 2: Table S8 for gene ontology results for circum-basmati-specific gene gains).

Many of these genic PAVs could have been important during the rice domestication process [11]. Gene deletions, in particular, are more likely to have a functional consequence than single-nucleotide polymorphisms or short indels and may underlie drastic phenotypic variation. In the context of crop domestication and diversification, this could have led to desirable phenotypes in human-created agricultural environments. For instance, several domestication phenotypes in rice are known to be caused by gene deletions [35, 62–66].

There were 873 gene orthogroups for which neither of the circum-basmati genomes had a gene member, but for which genomes for all three other rice variety groups (N22, Nipponbare, and R498) had at least one gene member. Among these, there were 545 orthogroups for which N22, Nipponbare, and R498 each had a single-copy gene member, suggesting that the deletion of these genes in both the Basmati 334 and Dom Sufid genomes could have had a major effect in circum-basmati. We aligned Illumina sequencing data from our circum-basmati population dataset to the japonica Nipponbare genome and calculated deletion frequencies of Nipponbare genes that belonged to the 545 orthogroups (see Additional file 2: Table S9 for gene deletion frequencies in the circum-basmati population for the Nipponbare genes that are missing in Basmati 334 and Dom Sufid). The vast majority of these Nipponbare genes (509 orthogroups or 93.4%) were entirely absent in the circum-basmati population, further indicating that these were circum-basmati-specific gene deletions fixed within this variety group.

One of the genes specifically deleted in circum-basmati rice varieties was *Awn3-1* (Os03g0418600), which was identified in a previous study as associated with altered awn length in japonica rice [67]. Reduced awn length is an important domestication trait that was selected for ease of harvesting and storing rice seeds [68]. This gene was missing in both circum-basmati genomes, and no region could be aligned to the Nipponbare *Awn3-1* genic region (Fig. 2c). Instead of the *Awn3-1* coding sequence, this genomic region contained an excess of transposable element sequences, suggesting an accumulation of repetitive DNA may have been involved in this gene’s deletion. The flanking arms upstream and downstream of Os03g0418600 were annotated in both circum-basmati genomes and were syntenic to the regions in both Nipponbare and N22. These flanking arms, however, were also accumulating transposable element sequences, indicating that this entire genomic region may be degenerating in both circum-basmati rice genomes.

We then examined the deletion status for other genes involved in the domestication of *O. sativa*. We focused on the genes that were previously implicated to be involved in the initial domestication phase of rice [11] where the genes were selected during the transformation of a wild rice into a domesticated rice—i.e., *Rc* (Os07g0211500) [19], *Bh4* (Os04g0460200) [69], *PROG1* (Os07g0153600) [70], *OsC1* (Os06g0205100) [71], *Sh4* (Os04g0670900) [72], *GS3* (Os03g0407400) [73], *qSH1* (Os01g0848400) [20], and *qSW5* (Os05g0187500) [62]. Our aim was to draw inferences on whether the domestication history of circum-basmati rice may have differed from that of the other rice subpopulations. Results showed none of these genes were deleted in the circum-basmati population (Additional file 2: Table S8). This suggests that unlike the domestication process of domesticated African rice (*O. glaberrima* [74]), gene deletions were not a major contributor during the initial domestication phase of circum-basmati rice. Its likely many of the gene deletions that were selected during the domestication of the circum-basmati rice occurred during the cultivation period [11], when culinary or cultural preferences have selected for unique circum-basmati specific traits.

### Repetitive DNA and retrotransposon dynamics in the circum-basmati genomes

**Fig. 4:**
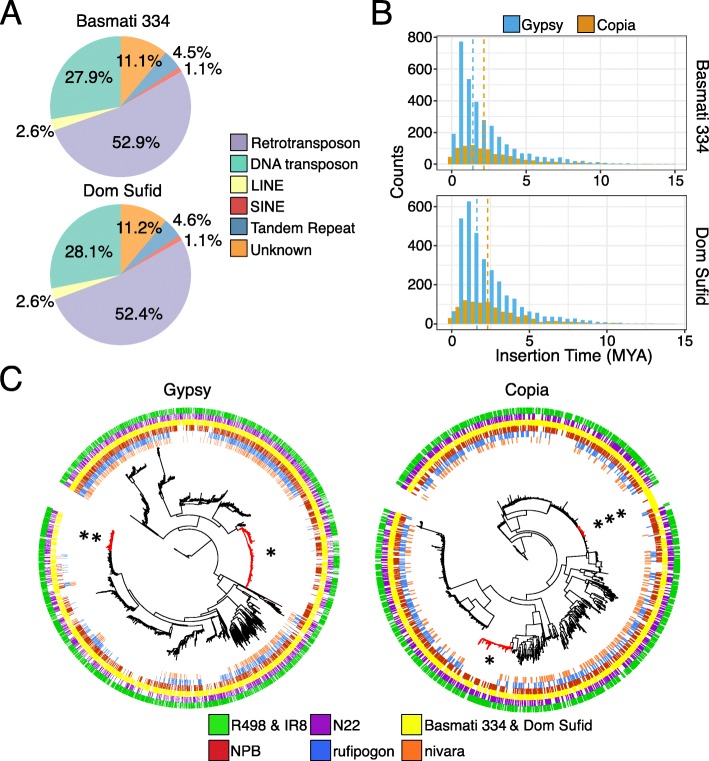
Repetitive DNA landscape of the Basmati 334 and Dom Sufid genomes. **a** Proportion of repetitive DNA content in the circum-basmati genomes represented by each repeat family. **b** Distribution of insert times for the *gypsy* and *copia* LTR retrotransposons. **c** Phylogeny of *gypsy* and *copia* LTR retrotransposons based on the *rve* gene. LTR retrotransposons were annotated from the reference genomes of domesticated and wild rice

Repetitive DNA makes up more than 44% of the Basmati 334 and Dom Sufid genome assemblies (Table 2). Consistent with genomes of other plant species [75], the repetitive DNA was largely composed of Class I retrotransposons, followed by Class II DNA transposons (Fig. 4a). In total, 171.1 Mb was annotated as repetitive for Basmati 334 and 169.5 Mb for Dom Sufid. The amount of repetitive DNA in the circum-basmati genomes was higher than that in the Nipponbare (160.6 Mb) and N22 genomes (152.1 Mb), but lower than that in the indica R498 (175.9 Mb) and IR8 (176.0 Mb) genomes. These differences in the total amount of repetitive DNA were similar to overall genome assembly size differences (Table 3), indicating that variation in repeat DNA accumulation is largely driving genome size differences in rice [76].

We focused our attention on retrotransposons, which made up the majority of the rice repetitive DNA landscape (Fig. 4a). Using *LTRharvest* [77, 78], we identified and de novo-annotated LTR retrotransposons in the circum-basmati genomes. *LTRharvest* annotated 5170 and 5150 candidate LTR retrotransposons in Basmati 334 and Dom Sufid, respectively (Additional file 2: Tables S10 and S11). Of these, 4180 retrotransposons (80.9% of all candidate LTR retrotransposons) in Basmati 334 and 4228 (82.1%) in Dom Sufid were classified as LTR retrotransposons by *RepeatMasker*’s *RepeatClassifer* tool (http://www.repeatmasker.org). Most LTR retrotransposons were from the *gypsy* and *copia* superfamilies [79, 80], which made up 77.1% (3225 *gypsy* elements) and 21.9% (915 *copia* elements) of LTR retrotransposons in the Basmati 334 genome, and 76.4% (3231 *gypsy* elements) and 22.8% (962 *copia* elements) of LTR retrotransposons in the Dom Sufid genome, respectively. Comparison of LTR retrotransposon content among reference genomes from different rice variety groups (Additional file 1: Figure S5) revealed that genomes assembled to near completion (i.e., Nipponbare, N22, Basmati 334, Dom Sufid, and indica varieties IR8 and R498, as well as MH63 and ZS97 [40]) had higher numbers of annotated retrotransposons than genomes generated from short-read sequencing data (GP295-1, circum-aus varieties DJ123 [38] and Kasalath [39], and indica variety IR64 [38]), suggesting genome assemblies from short-read sequencing data may be missing certain repetitive DNA regions.

Due to the proliferation mechanism of LTR transposons, the DNA divergence of an LTR sequence can be used to approximate the insertion time for an LTR retrotransposon [81]. Compared to other rice reference genomes, the insertion times for the Basmati 334 and Dom Sufid LTR retrotransposons were most similar to those observed for elements in the circum-aus N22 genome (Additional file 1: Figure S5). Within our circum-basmati assemblies, the *gypsy* superfamily elements had a younger average insertion time (~ 2.2 million years ago) than elements of the *copia* superfamily (~ 2.7 million years ago; Fig. 4b).

Concentrating on *gypsy* and *copia* elements with the *rve* (integrase; Pfam ID: PF00665) gene, we examined the evolutionary dynamics of these LTR retrotransposons by reconstructing their phylogenetic relationships across reference genomes for the four domesticated rice variety groups (N22, Basmati 334, Dom Sufid, R498, IR8, and Nipponbare), and the two wild rice species (*O. nivara* and *O. rufipogon*; Fig. 4c). The retrotransposons grouped into distinct phylogenetic clades, which likely reflect repeats belonging to the same family or subfamily [82]. The majority of phylogenetic clades displayed short external and long internal branches, consistent with rapid recent bursts of transposition observed across various rice LTR retrotransposon families [83].

The *gypsy* and *copia* superfamilies each contained a clade in which the majority of elements originated within *O. sativa*, and only present among the four domesticated rice variety groups (Fig. 4c, single star; see Additional files 2: Tables S12 and S13 for their genome coordinates). Elements in the *gypsy* superfamily phylogenetic clade had sequence similarity (963 out of the 1837 retrotransposons) to elements of the *hopi* family [84], while elements in the *copia* superfamily phylogenetic clade had sequence similarity (88 out of the 264) to elements in the *osr4* family [85]. Elements of the *hopi* family are found in high copy number in genomes of domesticated rice varieties [86] and this amplification has happened recently [87].

Several retrotransposon clades were restricted to certain rice variety groups. The *gypsy* superfamily harbored a phylogenetic clade whose elements were only present in genomes of circum-aus, circum-basmati, and indica varieties (Fig. 4c, double star; see Additional file 2: Table S14 for their genome coordinates), while we observed a clade comprised mostly of circum-basmati-specific elements within the *copia* superfamily (Fig. 4c, triple star; see Additional file 2: Table S15 for their genome coordinates). Only a few members of the *gypsy*-like clade had sequence similarity (7 out of 478) to elements of the *rire3* [88] and *rn215* [89] families. Members of both families are known to be present in high copy numbers in genomes of domesticated rice varieties, but their abundance differs between the japonica and indica variety groups [86], suggesting a *rire3*- or *rn215*-like element expansion in the circum-aus, circum-basmati, and indica genomes. A majority of the circum-basmati-specific *copia*-like elements had sequence similarity (109 out of 113) to members of the *houba* family [84], which are found in high copy numbers in certain individuals, but in lower frequency across the rice population [86]. This suggests the *houba* family might have undergone a recent expansion specifically within the circum-basmati genomes.

### Phylogenomic analysis on the origins of circum-basmati rice

**Fig. 5:**
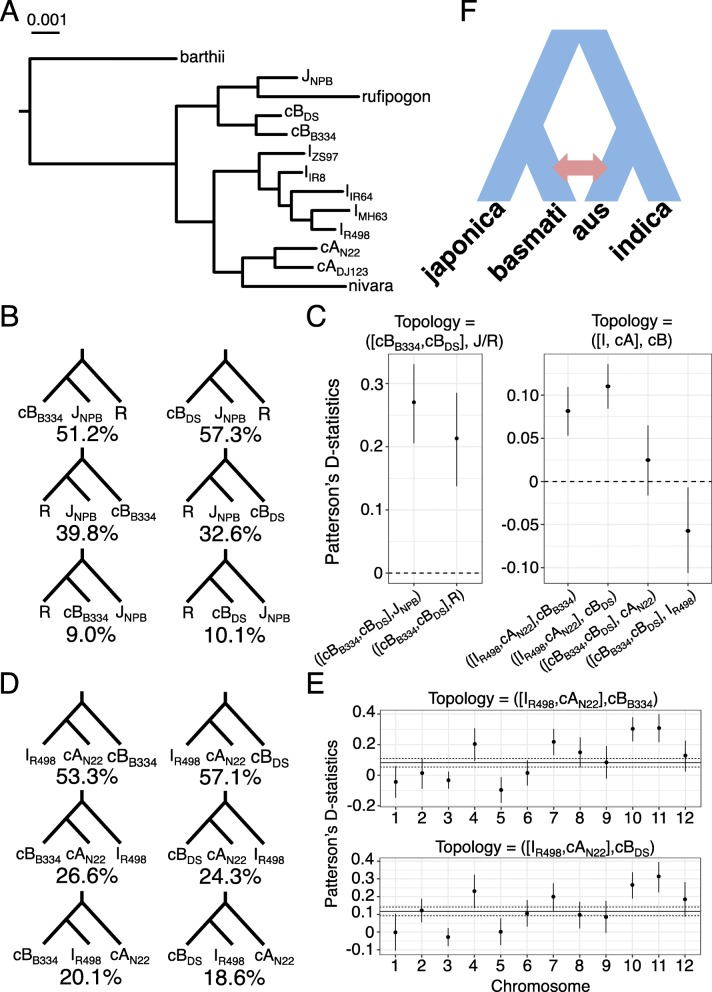
Comparative genomic analysis of circum-basmati rice evolution. The *Oryza sativa* variety groups are labeled as circum-aus (cA), circum-basmati (cB), indica (I), and japonica (J), and the wild relative is *O. rufipogon* (R). **a** Maximum likelihood tree based on fourfold degenerate sites. All nodes had over 95% bootstrap support. **b** Percentage of genes supporting the topology involving japonica Nipponbare, circum-basmati Basmati 334 (B334) and Dom Sufid (DS), and *O. rufipogon* after an Approximately Unbiased (AU) test. **c** Results of ABBA-BABA tests. Shown are median Patterson’s D-statistics with 95% confidence intervals determined from a bootstrapping procedure. For each tested topology, the outgroup was always *O. barthii*. **d** Percentage of genes supporting the topology involving circum-aus N22, circum-basmati, and indica R498 after an AU test. **e** Per-chromosome distribution of D-statistics for the trio involving R498, N22, and each circum-basmati genome. Genome-wide D-statistics with 95% bootstrap confidence intervals are indicated by the dark and dotted lines. **f** Model of admixture events that occurred within domesticated Asian rice. The direction of admixture has been left ambiguous, as the ABBA-BABA test cannot detect the direction of gene flow

We estimated the phylogenetic relationships within and between variety groups of domesticated Asian rice. Our maximum likelihood phylogenetic tree, based on fourfold degenerate sites from the Nipponbare coding sequences (Fig. 5a), showed that each cultivar was monophyletic with respect to its variety group of origin. In addition, the circum-basmati group was sister to japonica rice, while the circum-aus group was sister to indica. Consistent with previous observations, the wild rice *O. nivara* and *O. rufipogon* were sister to the circum-aus and japonica rice, respectively [14]. While this suggests that each domesticated rice variety group may have had independent wild progenitors of origin, it should be noted that recent hybridization between wild and domesticated rice [90, 91] could lead to similar phylogenetic relationships.

To further investigate phylogenetic relationships between circum-basmati and japonica, we examined phylogenetic topologies of each gene involving the trio Basmati 334, Nipponbare, and *O. rufipogon*. For each gene, we tested which of three possible topologies for a rooted three-species tree—i.e., [(P1, P2), P3], O, where O is outgroup *O. barthii* and P1, P2, and P3 are Basmati 334 (or Dom Sufid), Nipponbare, and *O. rufipogon*, respectively—were found in the highest proportion. For the trio involving Basmati 334, Nipponbare, and *O. rufipogon*, there were 7581 genes (or 32.6%), and for the trio involving Dom Sufid, Nipponbare, and *O. rufipogon*, there were 7690 genes (or 33.1%), which significantly rejected one topology over the other two using an Approximately Unbiased (AU) topology test [92]. In both trios, the majority of those genes supported a topology that grouped circum-basmati and Nipponbare as sister to each other (Fig. 5b; 3881 [or 51.2%] and 4407 [or 57.3%] genes for Basmati 334 and Dom Sufid, respectively). A lower number of genes (3018 [or 39.8%] and 2508 [or 32.6%] genes for Basmati 334 and Dom Sufid, respectively) supported the topology that placed Nipponbare and *O. rufipogon* together.

Our initial topology test suggested that the trio involving Dom Sufid, Nipponbare, and *O. rufipogon* had a higher proportion of genes supporting the [(circum-basmati, japonica), *O. rufipogon*] topology compared to the trio involving Basmati 334, Nipponbare, and *O. rufipogon* (Fig. 5b). This suggested within-population variation in the amount of japonica or *O. rufipogon* ancestry across the circum-basmati genomes due to differences in gene flow. To test for introgression, we employed D-statistics from the ABBA-BABA test [93, 94]. We conducted ABBA-BABA tests involving the topology [(Basmati 334, Dom Sufid), Nipponbare or *O. rufipogon*] to examine the differences in introgression between the circum-basmati and japonica or *O. rufipogon* genomes. The results showed significantly positive D-statistics for the topology [(Basmati 334, Dom Sufid), Nipponbare] (Fig. 5c left panel; *z*-score = 8.42 and *D* = 0.27 ± 0.032), indicating that Dom Sufid shared more alleles with japonica than Basmati 334 did due to a history of more admixture with japonica. The D-statistics involving the topology [(Basmati 334, Dom Sufid), *O. rufipogon*] were also significantly positive (Fig. 5c left panel; *z*-score = 5.57 and *D* = 0.21 ± 0.038).

### Signatures of admixture between circum-basmati and circum-aus rice genomes

Due to extensive admixture between rice variety group genomes [14], we examined whether the basmati genome was also influenced by gene flow with other divergent rice variety groups (i.e., circum-aus or indica rice). A topology test was conducted for a rooted, three-population species tree. For the trio involving Basmati 334, circum-aus variety N22, and indica variety R498, there were 7859 genes (or 35.3%), and for the trio involving Dom Sufid, N22, and R498, there were 8109 genes (or 37.8%), which significantly rejected one topology over the other two after an AU test. In both trios, more than half of the genes supported the topology grouping circum-aus and indica as sisters (Fig. 5d). In addition, more genes supported the topology grouping circum-aus and circum-basmati as sisters than the topology grouping indica and circum-basmati as sisters. This suggested that the circum-aus variety group might have contributed a larger proportion of genes to circum-basmati through gene flow than the indica variety group did.

To test for evidence of admixture, we conducted ABBA-BABA tests involving trios of the circum-basmati, N22, and R498 genomes. Results showed significant evidence of gene flow between circum-aus and both circum-basmati genomes—Fig. 5c, right panel; *z*-score = 5.70 and *D* = 0.082 ± 0.014 for topology [(R498, N22), Basmati 334]; and *z*-score = 8.44 and *D* = 0.11 ± 0.013 for topology [(R498, N22), Dom Sufid]. To test whether there was variability in the circum-aus or indica ancestry in each of the circum-basmati genomes, we conducted ABBA-BABA tests for the topology [(Basmati 334, Dom Sufid), N22 or R498]. Neither of the ABBA-BABA tests involving the topology [(Basmati 334, Dom Sufid), N22] (Fig. 5c, right panel; *z*-score = 1.20 and *D* = 0.025 ± 0.021) or the topology [(Basmati 334, Dom Sufid), R498] (Fig. 5c, right panel; *z*-score = − 2.24 and *D* = − 0.06 ± 0.026) was significant, suggesting the amount of admixture from circum-aus to each of the two circum-basmati genomes was similar.

Because of the significant amount of admixture occurring between the circum-aus and circum-basmatigenomes, we examined whether this had affected the topology analysis involving the trio japonica, circum-basmati, and *O. rufipogon* (Fig. 5b). Specifically, we assessed whether the grouping of japonica and *O. rufipogon* as sister species (Fig. 5a) was an evolutionary artifact due to sharing of alleles between circum-basmati and circum-aus through admixture. We examined this by conducting the AU test on the four populations involving circum-aus, circum-basmati (Basmati 334 or Dom Sufid), japonica, and *O. rufipogon*, testing which of the 15 possible topologies for a rooted four-population sample (see Additional file 1: Figure S6 for the 15 topologies tested) was the best fit for each gene. Results showed there were 2774 genes involving Basmati 334 and 2665 genes involving Dom Sufid where the AU test significantly rejected one topology over the other 14 topologies (Additional file 1: Figure S6). The most frequent topology (> 30% of the genes) was one that both grouped japonica and *O. rufipogon* as sisters and grouped circum-basmati and circum-aus as sisters, which is a topology that occurs when there is admixture occurring between circum-basmati and circum-aus. The second most frequent topology (> 20% of the genes) was the species phylogeny (i.e., [(circum-basmati, japonica), *O. rufipogon*]) and this was represented fivefold higher than the remaining 13 topologies. In the end, this result partially explains the discrepancy between the genome-wide tree topology (Fig. 5a) and the gene-specific tree topology (Fig. 5b). The admixture occurring between circum-basmati and circum-aus had led to the spurious genome-wide topological relationship.

In sum, the phylogenomic analysis indicated that circum-basmati and japonica share the most recent common ancestor, while circum-aus has admixed with circum-basmati during its evolutionary history (Fig. 5f). We then examined whether admixture from circum-aus had affected each of the circum-basmati chromosomes to a similar degree. For both circum-basmati genomes, most chromosomes had D-statistics that were not different from the genome-wide D-statistics value or from zero (Fig. 5e). Exceptions were chromosomes 10 and 11, where the bootstrap D-statistics were significantly higher than the genome-wide estimate.

### Population analysis on the origin of circum-basmati rice

Since our analysis was based on single representative genomes from each rice variety group, we compared the results of our phylogenomic analyses to population genomic patterns in an expanded set of rice varieties from different groups. We obtained high coverage (> 14×) genomic re-sequencing data (generated with Illumina short-read sequencing) from landrace varieties in the 3K Rice Genome Project [7] and from circum-basmati rice landraces we re-sequenced. In total, we analyzed 24 circum-aus, 18 circum-basmati, and 37 tropical japonica landraces (see Additional file 2: Table S16 for variety names). The raw Illumina sequencing reads were aligned to the scaffolded Basmati 334 genome and computationally genotyped. A total of 4,594,290 polymorphic sites were called across the three rice variety groups and used for further analysis.

**Fig. 6:**
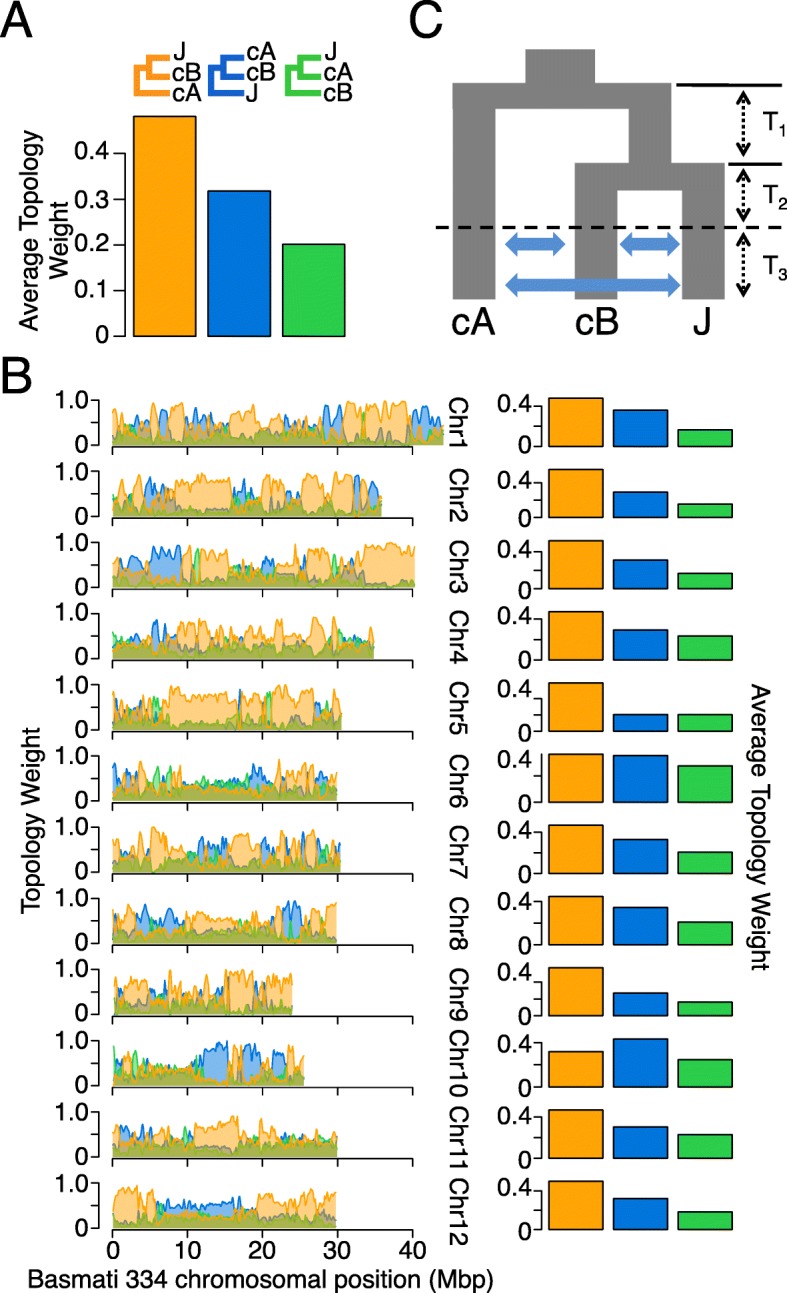
Population relationships among the circum-aus (cA), circum-basmati (cB), and japonica rice (J). **a** Sum of genome-wide topology weights for a three-population topology involving trios of the circum-aus, circum-basmati, and japonica rice. Topology weights were estimated across windows with 100 SNPs. **b** Chromosomal distributions of topology weights involving trios of the circum-aus, circum-basmati, and japonica rice (left), and the sum of the topology weights (right). **c** Best-fitting *δaδi* model for the circum-aus, circum-basmati, and japonica rice. See Additional file 2: Table S17 for parameter estimates

To quantify relationships between circum-aus, circum-basmati, and japonica, we conducted a topology-weighting analysis [95]. For three populations, there are three possible topologies and we conducted localized sliding window analysis to quantify the number of unique sub-trees that supported each tree topology. Consistent with the phylogenomic analysis results, the topology weight was the largest for the topology that grouped japonica and circum-basmati as sisters (Fig. 6a; topology weight = 0.481 with 95% confidence interval [0.479–0.483]). The topology that grouped circum-aus and circum-basmati together as sisters weighed significantly more (topology weight = 0.318 with 95% confidence interval [0.316–0.320]) than the topology that grouped japonica and circum-aus as sisters (topology weight = 0.201 with 95% confidence interval [0.199–0.203]). This was consistent with the admixture results from the comparative phylogenomic analysis, which detected evidence of gene flow between circum-aus and circum-basmati.

A treemix analysis was conducted for the three domesticated rice population (circum-aus, circum-basmati, and japonica) alongside the wild rice *O. rufipogon* and *O. barthii* (Additional file 1: Figure S7). We fitted zero to three migration edges in the model, and at three migration edges, the model log-likelihood started plateauing (Additional file 1: Figure S7B). At three migration edges, a migration edge was fitted between circum-aus and circum-basmati (Additional file 1: Figure S7A), consistent with our previous results. In addition, there were migration edges fitted between the wild rice *O. rufipogon* and circum-basmati and between the wild rice *O. barthii* and japonica. Overall, these migration results were consistent with recent studies that have documented the occurrence of admixture between wild and domesticated rice populations [74, 90, 91].

We then examined topology weights for each individual chromosome, since the ABBA-BABA tests using the genome assemblies had detected variation in circum-aus ancestry between different chromosomes (Fig. 5e). The results showed that for most of the chromosomes the topology [(japonica, circum-basmati), circum-aus] always weighed more than the remaining two topologies. An exception was observed for chromosome 10 where the topology weight grouping circum-aus and circum-basmati as sisters was significantly higher (topology weight = 0.433 with 95% confidence interval [0.424–0.442]) than the weight for the genome-wide topology that grouped japonica and circum-basmati as sisters (topology weight = 0.320 with 95% confidence interval [0.312–0.328]). This change in predominant topology was still observed when the weights were calculated across wider local windows (Additional file 1: Figure S8). Another exception could be seen for chromosome 6 where the genome-wide topology [(japonica, circum-basmati), circum-aus] (topology weight = 0.367 with 95% confidence interval [0.359–0.374]) and the admixture topology [(circum-aus, circum-basmati), japonica] (topology weight = 0.355 with 95% confidence interval [0.349–0.362]) had almost equal weights. In larger window sizes, the weight of the admixed topology was slightly higher than that of the genome-wide topology (Additional file 1: Figure S8).

To estimate the evolutionary/domestication scenario that might explain the observed relationships between the circum-aus, circum-basmati, and japonica groups, we used the diffusion-based approach of the program *δaδi* [96] and fitted specific demographic models to the observed allele frequency spectra for the three rice variety groups. Because all three rice groups have evidence of admixture with each other [7, 9, 14, 16], we examined 13 demographic scenarios involving symmetric, asymmetric, and “no migration” models between variety groups, with and without recent population size changes (Additional file 1: Figure S9). To minimize the effect of genetic linkage on the demography estimation, polymorphic sites were randomly pruned in 200-kb windows, resulting in 1918 segregating sites. The best-fitting demographic scenario was one that modeled a period of lineage splitting and isolation, while gene flow only occurred after formation of the three populations and at a later time (Fig. 6c; visualizations of the 2D site frequency spectrum and model fit can be seen in Additional file 1: Figure S10). This best-fitting model was one of the lesser-parameterized models we tested, and the difference in Akaike Information Criterion (ΔAIC) with the model with the second-highest likelihood was 25.46 (see Additional file 2: Table S17 for parameter estimates and maximum likelihood estimates for each demographic model).

### Genetic structure within the circum-basmati group

We used the circum-basmati population genomic data for the 78 varieties aligned to the scaffolded Basmati 334 genome and called the polymorphic sites segregating within this variety group. After filtering, a total of 4,430,322 SNPs across the circum-basmati dataset remained, which were used to examine population genetic relationships within circum-basmati.

**Fig. 7:**
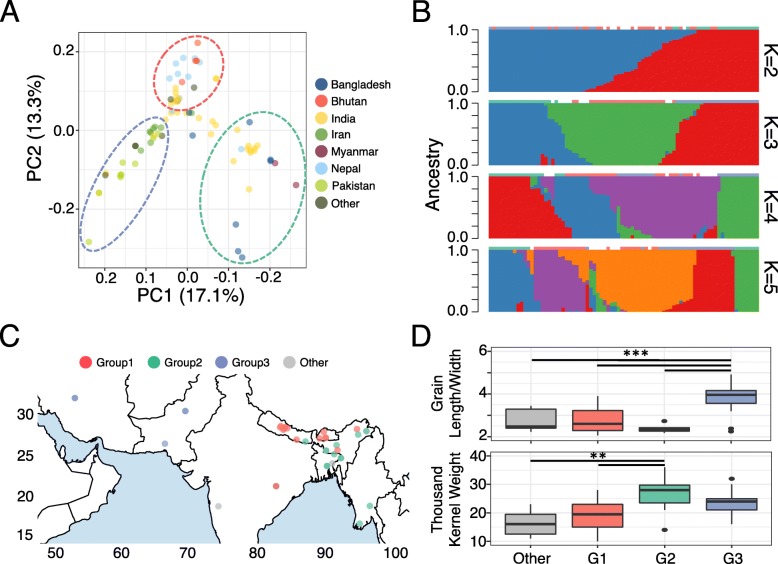
Population structure within the circum-basmati rice. **a** PCA plot for the 78-variety circum-basmati rice population genomic dataset. The three genetic groups designated by this study can be seen in the color-coded circles with dashed lines. **b** Proportion of ancestry plot for *K* = 2, 3, 4, and 5 across the 78 circum-basmati rice varieties. The color-coding from (**a**) is indicated above each sample’s ancestry proportion. **c** Geographic distribution of the 78 circum-basmati rice varieties with their grouping status color-coded according to **a**. **d** Agronomic measurements for the 78 circum-basmati rice varieties sorted into the three groups designated by this study. Two asterisks indicate *p* value < 0.01 and three asterisks indicate *p* value < 0.001

We conducted principal component analysis (PCA) using the polymorphism data and color-coded each circum-basmati rice variety according to its country of origin (Fig. 7a). The PCA suggested that circum-basmati rice could be divided into three major groups with clear geographic associations: (group 1) a largely Bhutan/Nepal-based group, (group 2) an India/Bangladesh/Myanmar-based group, and (group 3) an Iran/Pakistan-based group. The rice varieties that could not be grouped occupied an ambiguous space across the principal components, suggesting these might represent admixed rice varieties.

To obtain better insight into the ancestry of each rice variety, we used *fastSTRUCTURE* [97] and varied assumed ancestral population (*K*) from 2 to 5 groups so the ancestry proportion of each rice variety could be estimated (Fig. 7b). At *K* = 2, the India/Bangladesh/Myanmar and Iran/Pakistan rice groups were shown to have distinct ancestral components, while the Bhutan/Nepal group was largely an admixture of the other two groups. At *K* = 3, the grouping status designated from the PCA was largely concordant with the ancestral components. At *K* = 4, most India/Bangladesh/Myanmar rice had a single ancestral component, but Iran/Pakistan rice had two ancestral components that were shared with several Bhutan/Nepal landraces. Furthermore, several of the cultivars from the latter group seemed to form an admixed group with India/Bangladesh/Myanmar varieties. In fact, when a phylogenetic tree was reconstructed using the polymorphic sites, varieties within the India/Bangladesh/Myanmar and Iran/Pakistan groups formed a monophyletic clade with each other. On the other hand, Bhutan/Nepal varieties formed a paraphyletic group where several clustered with the Iran/Pakistan varieties (Additional file 1: Figure S11).

We then conducted a second *fastSTRUCTURE* analysis on the circum-basmati population, this time including the japonica and circum-aus populations while varying *K* from 2 to 5 groups (Additional file 1: Figure S12). From *K* = 2 to 5, the japonica and circum-aus groups always formed two distinct genetic groups. At *K* = 5, the three circum*-*basmati genetic groups that were designated in the first analysis (Fig. 7) were still observed across the circum-basmati population. In lower *K*, we see that the different circum-basmati genetic groups had differing amounts of japonica or circum-aus ancestry. Specifically, the Iran/Pakistan group had more circum-aus ancestry, while the India/Bangladesh/Myanmar group had more japonica ancestry. The Bhutan/Nepal group again was suggested to be an admixture of the other two circum-basmati rice genetic groups.

In summary, the circum-basmati rice has evolved across a geographic gradient with at least three genetic groups (Fig. 7c). These existed as distinct ancestral groups that later admixed to form several other circum-basmati varieties. Group 1 and group 3 rice in particular may have experienced greater admixture, while the group 2 landraces remained genetically more isolated from other circum-basmati subpopulations. We also found differences in agronomic traits associated with our designated groups (Fig. 7d). The grain length to width ratio, which is a highly prized trait in certain circum-basmati rice [24], was significantly larger in group 3 Iran/Pakistan varieties. The thousand-kernel weights, on the other hand, were highest for group 2 India/Bangladesh/Myanmar varieties and were significantly higher than those for the ungrouped and group 1 Bhutan/Nepal varieties.

## Discussion

Nanopore sequencing is becoming an increasingly popular approach to sequence and assemble the often large and complex genomes of plants [98–100]. Here, using long-read sequences generated with Oxford Nanopore Technologies’ sequencing platform, we assembled genomes of two circum-basmati rice cultivars, with quality metrics that were comparable to other rice variety group reference genome assemblies [37, 40, 41]. With modest genome coverage, we were able to develop reference genome assemblies that represented a significant improvement over a previous circum-basmati reference genome sequence, which had been assembled with a > 3-fold higher genome coverage than ours, but from short-read sequences [42]. With additional short-read sequencing reads, we were able to correct errors from the nanopore sequencing reads, resulting in two high-quality circum-basmati genome assemblies.

Even with long-read sequence data, developing good plant reference genome sequences still requires additional technologies such as optical mapping or Hi-C sequencing for improving assembly contiguity [101–104], which can be error prone as well [56]. Our assemblies were also fragmented into multiple contigs, but sizes of these contigs were sufficiently large that we could use reference genome sequences from another rice variety group to anchor the majority of contigs and scaffold them to higher-order chromosome-level assemblies. Hence, with a highly contiguous draft genome assembly, reference genome-based scaffolding can be a cost-efficient and powerful method of generating chromosome-level assemblies.

Repetitive DNA constitutes large proportions of plant genomes [105], and there is an advantage to using long-read sequences for genome assembly as it enables better annotation of transposable elements. Many transposable element insertions have evolutionarily deleterious consequences in the rice genome [54, 106, 107], but some insertions could have beneficial effects on the host [108]. Using our genome assembly, we have identified retrotransposon families that have expanded specifically within circum-basmati genomes. While more study will be necessary to understand the functional effects of these insertions, long-read sequences have greatly improved the assembly and identification of repeat types.

Due to a lack of archaeobotanical data, the origins of circum-basmati rice have remained elusive. Studies of this variety group’s origins have primarily focused on genetic differences that exist between circum-basmati and other Asian rice variety groups [6, 7]. Recently, a study suggested that circum-basmati rice (called “aromatic” in that study) was a product of hybridization between the circum-aus and japonica rice variety groups [17]. This inference was based on observations of phylogenetic relationships across genomic regions that showed evidence of domestication-related selective sweeps. These regions mostly grouped circum-basmati with japonica or circum-aus. In addition, chloroplast haplotype analysis indicated that most circum-basmati varieties carried a chloroplast derived from a wild rice most closely related to circum-aus landraces [109]. Our evolutionary analysis of circum-basmati rice genomes generally supported this view. Although our results suggest that circum-basmati had its origins primarily in japonica, we also find significant evidence of gene flow originating from circum-aus, which we detected both in comparative genomic and population genomic analyses. Our results are also consistent with the observation of genome-wide fine-scale admixture tracts that showed japonica rice contributing the highest amount of genetic material to circum-basmati, followed by circum-aus [110]. Demographic modeling indicated a period of isolation among circum-aus, circum-basmati, and japonica, with gene flow occurring only after lineage splitting of each group. Here, our model is consistent with the current view that gene flow is a key evolutionary process associated with the diversification of rice [10, 12–14, 16, 111, 112].

Interestingly, we found that chromosome 10 of circum-basmati had an evolutionary history that differed significantly from that of other chromosomes. Specifically, compared to japonica, this chromosome had the highest proportion of presence/absence variation and shared more alleles with circum-aus. Based on this result, we hypothesize that this is largely due to higher levels of introgression from circum-aus into chromosome 10 compared to other chromosomes. Such a deviation of evolutionary patterns on a single chromosome has been observed in the *Aquilegia* genus [113], but to our knowledge has not been observed elsewhere. Why this occurred is unclear at present, but it may be that selection has driven a higher proportion of circum-aus alleles into chromosome 10. Future work will be necessary to clarify the consequence of this higher level of admixture on chromosome 10.

Very little is known about population genomic diversity within circum-basmati. Our analysis suggests the existence of at least three genetic groups within this variety group, and these groups showed geographic structuring. Several varieties from group 1 (Bhutan/Nepal) and group 3 (Iran/Pakistan) had population genomic signatures consistent with an admixed population, while group 2 (India/Bangladesh/Myanmar) was genetically more distinct from the other two subpopulations. In addition, the geographic location of the India/Bangladesh/Myanmar group largely overlaps the region where circum-aus varieties were historically grown [114, 115]. Our genome-wide evidence of geographic structuring is also consistent with past studies that examined isozyme loci or simple sequence repeat (SSR) markers in Asian rice, and finding a longitudinal genetic structure from Iran to Myanmar. Isozymes could differentiate the circum-basmati rice from other Asian rice subpopulations (classified as group V in Glaszmann [8]) and the varieties from Iran were especially differentiated at two isozyme loci [116]. SSR markers differentiated the Myanmar group V varieties from the rest of south and west Asian varieties [117]. Based on this study and past evidences, genome-wide survey of an expanded sample of circum-basmati should assist in determining the fine-scale genetic structure of the circum-basmati population.

Given the extensive history of admixture that circum-basmati rice has with circum-aus, the India/Bangladesh/Myanmar group may have been influenced particularly strongly by gene flow from circum-aus. How these three genetic subpopulations were established may require a deeper sampling with in-depth analysis, but the geographically structured genomic variation shows that the diversity of circum-basmati has clearly been underappreciated. In addition, the Basmati 334 and Dom Sufid varieties, for which we generated genome assemblies in this study, both belong to the Iran/Pakistan genetic group. Thus, our study still leaves a gap in our knowledge of genomic variation in the Bhutan/Nepal and India/Bangladesh/Myanmar genetic groups, and varieties in these groups would be obvious next targets for generating additional genome assemblies.

## Conclusions

In conclusion, our study shows that generating high-quality plant genome assemblies is feasible with relatively modest amounts of resources and data. Using nanopore sequencing, we were able to produce contiguous, chromosome-level genome assemblies for cultivars in a rice variety group that contains economically and culturally important varieties. Our reference genome sequences have the potential to be important genomic resources for identifying single-nucleotide polymorphisms and larger structural variations that are unique to circum-basmati rice. Analyzing de novo genome assemblies for a larger sample of Asian rice will be important for uncovering and studying hidden population genomic variation too complex to study with only short-read sequencing technology.

## Materials and methods

### Plant material

Basmati 334 (IRGC 27819; GeneSys passport: https://purl.org/germplasm/id/23601903-f8c3-4642-a7fc-516a5bc154f7) is a basmati (sensu stricto) landrace from Pakistan and was originally donated to the International Rice Research Institute (IRRI) by the Agricultural Research Council (ARC) in Karachi (donor accession ID: PAK. SR. NO. 39). Dom Sufid (IRGC 117265; GeneSys passport: https://purl.org/germplasm/id/fb861458-09de-46c4-b9ca-f5c439822919) is a sadri landrace from Iran. Seeds from accessions IRGC 27819 and IRGC 117265 were obtained from the IRRI seed bank, surface-sterilized with bleach, and germinated in the dark on a wet paper towel for 4 days. Seedlings were transplanted individually in pots containing continuously wet soil in a greenhouse at New York University’s Center for Genomics and Systems Biology and cultivated under a 12-h day-12-h night photoperiod at 30 °C. Plants were kept in the dark in a growth cabinet under the same climatic conditions for 4 days prior to tissue harvesting. Continuous darkness induced chloroplast degradation, which diminishes the amount of chloroplast DNA that would otherwise end up in the DNA extracted from the leaves.

### DNA extractions

Thirty-six 100-mg samples (3.6 g total) of leaf tissue from a total of 10 one-month-old plants were flash-frozen at harvest for each accession and stored at − 80 °C. DNA extractions were performed by isolating the cell nuclei and gently lysing the nuclei to extract intact DNA molecules [118]. Yields ranged between 140 and 150 ng/μl.

### Library preparation and nanopore sequencing

Genomic DNA was visualized on an agarose gel to determine shearing. DNA was size-selected using BluePippin BLF7510 cassette (Sage Science) and high-pass mode (> 20 kb) and prepared using Oxford Nanopore Technologies’ standard ligation sequencing kit SQK-LSK109. FLO-MIN106 (R9.4) flowcells were used for sequencing on the GridION X5 platform.

### Library preparation and Illumina sequencing

Extracted genomic DNA was prepared for short-read sequencing using the Illumina Nextera DNA Library Preparation Kit. Sequencing was done on the Illumina HiSeq 2500 – HighOutput Mode v3 with 2 × 100 bp read configuration, at the New York University Genomics Core Facility.

### Genome assembly, polishing, and scaffolding

After completion of sequencing, the raw signal intensity data was used for base calling using *flip flop* (version 2.3.5) from Oxford Nanopore Technologies. Reads with a mean qscore (quality) greater than 8 and a read length greater than 8 kb were used and trimmed for adaptor sequences using *Porechop* (https://github.com/rrwick/Porechop). Raw nanopore sequencing reads were corrected using the program *Canu* [119] and then assembled with the genome assembler *Flye* [120].

The initial draft assemblies were polished for three rounds using the raw nanopore reads with *Racon* ver. 1.2.1 [121] and one round with *Medaka* (https://github.com/nanoporetech/medaka) from Oxford Nanopore Technologies. Afterwards, reads from Illumina sequencing were used by *bwa-mem* [122] to align to the draft genome assemblies. The alignment files were then used by *Pilon* ver. 1.22 [123] for three rounds of polishing.

Contigs were scaffolded using a reference genome-guided scaffolding approach implemented in *RaGOO* [56]. Using the Nipponbare genome as a reference, we aligned the circum-basmati genomes using *Minimap2* [124]. *RaGOO* was then used to order the assembly contigs. Space between contigs was artificially filled in with 100 “N” blocks.

Genome assembly statistics were calculated using the *bbmap stats.sh* script from the *BBTools* suite (https://jgi.doe.gov/data-and-tools/bbtools/). Completeness of the genome assemblies was evaluated using *BUSCO* ver. 2.0 [125]. Synteny between the circum-basmati genomes and the Nipponbare genome was visualized using *D-GENIES* [126]. Genome-wide dotplot from *D-GENIES* indicated the initial genome assembly of Dom Sufid had an evidence of a large chromosomal fusion between the ends of chromosome 4 and 10. Closer examination of this contig (named contig_28 of Dom Sufid) showed the break point overlapped the telomeric repeat sequence, indicating there had been a misassembly between the ends of chromosome 4 and 10. Hence, contig_28 was broken up into two so that each contig represented the respective chromosome of origin and was then subsequently scaffolded using *RaGOO*.

Inversions that were observed in the dot plot were computationally verified independently using raw nanopore reads. The long-read-aware aligner *ngmlr* [55] was used to align the nanopore reads to the Nipponbare genome, after which the long-read-aware structural variation caller *sniffles* [55] was used to call and detect inversions.

The number of sites aligning to the Nipponbare genome was determined using the *Mummer4* package [127]. Alignment delta files were analyzed with the *dnadiff* suite from the *Mummer4* package to calculate the number of aligned sites and the number of differences between the Nipponbare genome and the circum-basmati genomes.

### Gene annotation and analysis

Gene annotation was conducted using the *MAKER* program [52, 53]. An in-depth description of running *MAKER* can be found on the website: https://gist.github.com/darencard/bb1001ac1532dd4225b030cf0cd61ce2. We used published *Oryza* genic sequences as evidence for the gene modeling process. We downloaded the Nipponbare cDNA sequences from RAP-DB (https://rapdb.dna.affrc.go.jp/) to supply as EST evidence, while the protein sequences from the 13 *Oryza* species project [37] were used as protein evidence for the *MAKER* pipeline. Repetitive regions identified from the repeat analysis were used to mask out the repeat regions for this analysis. After a first round of running *MAKER*, the predicted genes were used by *SNAP* [128] and *Augustus* [129] to create a training dataset of gene models, which was then used for a second round of *MAKER* gene annotation. Orthology between the genes from different rice genomes was determined with *Orthofinder* ver. 1.1.9 [59]. Ortholog statuses were visualized with the *UpSetR* package [130].

Gene ontology for the orthogroups that are missing specifically in the circum-basmati was examined by using the japonica Nipponbare gene and conducting a gene ontology enrichment analysis on *agriGO* v2.0 [131]. Gene ontology enrichment analysis for the circum-basmati-specific orthogroups was conducted first by predicting the function and gene ontology of each circum-basmati genome gene model using the eggnog pipeline [132]. We required an ontology to have more than 10 genes as a member for further consideration, and enrichment was tested through a hypergeometric test using the *GOstat* package [133].

### Repetitive DNA annotation

The repeat content of each genome assembly was determined using *Repeatmasker* ver. 4.0.5 (http://www.repeatmasker.org/RMDownload.html). We used the *Oryza*-specific repeat sequences that were identified from Choi et al. [14] (DOI: 10.5061/dryad.7cr0q), who had used *Repeatmodeler* ver. 1.0.8 (http://www.repeatmasker.org/RepeatModeler.html) to de novo-annotate repetitive elements across wild and domesticated *Oryza* genomes [37].

LTR retrotransposons were annotated using the program *LTRharvest* [134] with parameters adapted from [135]. LTR retrotransposons were classified into superfamilies [82] using the program *RepeatClassifier* from the *RepeatModeler* suite. Annotated LTR retrotransposons were further classified into specific families using the 242 consensus sequences of LTR-RTs from the RetrOryza database [89]. We used *blastn* [136] to search the RetrOryza sequences, and each of our candidate LTR retrotransposons was identified using the “80-80-80” rule [82]: two TEs belong to the same family if they were 80% identical over at least 80 bp and 80% of their length.

Insertion times for the LTR retrotransposons were estimated using the DNA divergence between pairs of LTR sequences [81]. The L-INS-I algorithm in the alignment program *MAFFT* ver. 7.154b [137] was used to align the LTR sequences. *PAML* ver. 4.8 [138] was used to estimate the DNA divergence between the LTR sequences with the Kimura-2-parameter base substitution model [139]. DNA divergence was converted to divergence time (i.e., time since the insertion of a LTR retrotransposon) approximating a base substitution rate of 1.3 × 10^−8^ [140], which is two times higher than the synonymous site substitution rate.

### Presence/absence variation detection

PAVs between the Nipponbare genome and the circum-basmati assemblies were detected using the *Assemblytics* suites [60]. Initially, the Nipponbare genome was used as the reference to align the circum-basmati assemblies using the program *Minimap2*. The resulting SAM files were converted to files in delta format using the *sam2delta.py* script from the *RaGOO* suite. The delta files were then uploaded onto the online *Assemblytics* analysis pipeline (http://assemblytics.com/). Repetitive regions would cause multiple regions in the Nipponbare or circum-basmati genomes to align to one another, and in that case, *Assemblytics* would call the same region as a PAV multiple times. Hence, any PAV regions that overlapped for at least 70% of their genomic coordinates were collapsed to a single region.

The combination of *ngmlr* and *sniffles* was also used to detect the PAVs that differed between the Nipponbare genome and the raw nanopore reads for the circum-basmati rice. Because *Assemblytics* only detects PAVs in the range of 50 to 100,000 bp, we used this window as a size limit to filter out the PAVs called by *sniffles*. Only PAVs supported by more than 5 reads by *sniffles* were analyzed.

*Assemblytics* and *sniffles* call the breakpoints of PAVs differently. *Assemblytics* calls a single-best breakpoint based on the genome alignment, while *sniffles* calls a breakpoint across a predicted interval. To find overlapping PAVs between *Assemblytics* and *sniffles*, we added 500 bp upstream and downstream of the *Assemblytics*-predicted breakpoint positions.

### Detecting gene deletions across the circum*-*basmati population

Genome-wide deletion frequencies of each gene were estimated using the 78-variety circum-basmati population genomic dataset. For each of the 78 varieties, raw sequencing reads were aligned to the circum-basmati and Nipponbare genomes using *bwa-mem*. Genome coverage per site was calculated using *bedtools genomecov* [141]. For each variety, the average read coverage was calculated for each gene, and a gene was designated as deleted if its average coverage was less than 0.05×.

### Whole-genome alignment of *Oryza* genomes assembled de novo

Several genomes from published studies that were assembled de novo were analyzed. These include domesticated Asian rice genomes from the japonica variety group cv. Nipponbare [33]; the indica variety group cvs. 93-11 [32], IR8 [37], IR64 [38], MH63 [40], R498 [41], and ZS97 [40]; the circum-aus variety group cvs. DJ123 [38], Kasalath [39], and N22 [37]; and the circum-basmati variety group cv. GP295-1 [42]. Three genomes from wild rice species were also analyzed; these were *O. barthii* [35], *O. nivara* [37], and *O. rufipogon* [37].

Alignment of the genomes assembled de novo was conducted using the approach outlined in Haudry et al. [142], and this alignment approach has been used in another rice comparative genomic study [14]. Briefly, this involved using the Nipponbare genome as the reference for aligning all other genome assemblies. Alignment between japonica and a query genome was conducted using *LASTZ* ver. 1.03.73 [143], and the alignment blocks were chained together using the UCSC Kent utilities [144]. For japonica genomic regions with multiple chains, the chain with the highest alignment score was chosen as the single-most orthologous region. This analyzes only one of the multiple regions that are potentially paralogous between the japonica and query genomes, but this was not expected to affect the downstream phylogenomic analysis of determining the origin and evolution of the circum*-*basmati rice variety group. All pairwise genome alignments between the japonica and query genomes were combined into a multi-genome alignment using *MULTIZ* [145].

### Phylogenomic analysis

The multi-genome alignment was used to reconstruct the phylogenetic relationships between the domesticated and wild rice. Fourfold degenerate sites based on the gene model of the reference japonica genome were extracted using the *msa_view* program from the *phast* package ver. 1.4 [146]. The fourfold degenerate sites were used by *RAxML* ver. 8.2.5 [147] to build a maximum likelihood-based tree, using a general time-reversible DNA substitution model with gamma-distributed rate variation.

To investigate the genome-wide landscape of introgression and incomplete lineage sorting, we examined the phylogenetic topologies of each gene [148]. For a three-species phylogeny using *O. barthii* as an outgroup, there are three possible topologies. For each gene, topology-testing methods [149] can be used to determine which topology significantly fits the gene of interest [14]. *RAxML*-estimated site-likelihood values were calculated for each gene and the significant topology was determined using the Approximately Unbiased (AU) test [92] from the program *CONSEL* v. 0.20 [150]. Genes with AU test results with a likelihood difference of 0 were omitted, and the topology with an AU test support of greater than 0.95 was selected.

### Testing for evidence of admixture

Evidence of admixture between variety groups was detected using the ABBA-BABA test D-statistics [93, 94]. In a rooted three-taxon phylogeny [i.e., “((P1,P2),P3),O” where P1, P2, and P3 are the variety groups of interest and O is outgroup *O. barthii*], admixture can be inferred from the combination of ancestral (“A”) and derived (“B”) allelic states of each individual. The ABBA conformation arises when variety groups P2 and P3 share derived alleles, while the BABA conformation is found when P1 and P3 share derived alleles. The difference in the frequency of the ABBA and BABA conformations is measured by the D-statistics, where significantly positive D-statistics indicate admixture between the P2 and P3 variety groups, and significantly negative D-statistics indicate admixture between the P1 and P3 variety groups. The genome was divided into 100,000-bp bins for jackknife resampling and calculation of the standard errors. The significance of the D-statistics was calculated using the *Z*-test, and D-statistics with *z*-scores greater than |3.9| (*p* < 0.0001) were considered significant.

### Population genomic analysis

We downloaded FASTQ files from the 3K Rice Genome Project [7] for rice varieties that were determined to be circum-basmati varieties in that project. An additional 8 circum-basmati varieties were sequenced on the Illumina sequencing platform as part of this study. The raw reads were aligned to the scaffolded Basmati 334 genome using the program *bwa-mem*. PCR duplicates were determined computationally and removed using the program *picard* version 2.9.0 (http://broadinstitute.github.io/picard/). Genotype calls for each site were conducted using the *GATK HaplotypeCaller* engine using the option “-ERC GVCF.” The output files were in the genomic variant call format (gVCF), and the gVCFs from each variety were merged using the *GATK GenotypeGVCFs* engine.

SNP and INDEL variants from the population variant file were filtered independently using the *GATK* bestpractice hard filter pipeline [151]. SNP variants within 5 bps of an INDEL variant were filtered. *Vcftools* version 0.1.15 [152] was used to filter sites for which genotypes were not called for more than 20% of the varieties. Because domesticated rice is an inbreeding species, we also implemented a heterozygosity filter by filtering out sites that had a heterozygote genotype in more than 5% of the samples using the program *vcffilterjdk.jar* from the *jvarkit* suite (https://figshare.com/articles/JVarkit_java_based_utilities_for_Bioinformatics/1425030). Missing genotypes were imputed and phased using *Beagle* version 4.1 [153].

To examine the within-circum-basmati variety group population structure, we first randomly pruned the sites by sampling a polymorphic site every 200,000 bp using *plink* [154]. *Plink* was also used to conduct a principal component analysis. Ancestry proportions of each sample were estimated using *fastSTRUCTURE* [97]. A neighbor-joining tree was built by calculating the pairwise genetic distances between samples using the Kronecker delta function-based equation [155]. From the genetic distance matrix, a neighbor-joining tree was built using the program *FastME* [156].

### Evolutionary relationships among the circum*-*basmati, circum*-*aus, and japonica populations

To investigate the evolutionary origins of the circum-basmati population, we focused on the landrace varieties that had been sequenced with a genome-wide coverage of greater than 14×. The population data for the circum-aus and japonica populations were obtained from the 3K Rice Genome Project [7], from which we also analyzed only the landrace varieties that had been sequenced with a genome-wide coverage greater than 14×. For an outgroup, we obtained *O. barthii* sequencing data from previous studies [35, 74] and focused on the samples that were not likely to be feralized rice [74]. The Illumina reads were aligned to the scaffolded Basmati 334 genome and SNPs were called and filtered according to the procedure outlined in the “Population genomic analysis” section.

We examined the genome-wide local topological relationship using *twisst* [95]. Initially, a sliding window analysis was conducted to estimate the local phylogenetic trees in windows with a size of 100 or 500 polymorphic sites using *RAxML* with the GTRCAT substitution model. The script *raxml_sliding_windows.py* from the *genomics_general* package by Simon Martin (https://github.com/simonhmartin/genomics_general/tree/master/phylo) was used. The “complete” option of *twisst* was used to calculate the exact weighting of each local window.

### Treemix analysis

A past study by Wang et al. [90] had found evidence of admixture between domesticated rice and wild rice in a dataset of genome sequences from a global sample of rice [16]. Therefore, we obtained *O. rufipogon* genome data from a separate study that sequenced five samples to a high coverage (> 10×) [157]. The *O. rufipogon* population sample was combined with the population genomic dataset from the section “Evolutionary relationships among the circum*-*basmati, circum*-*aus, and japonica populations.” Polymorphic sites were randomly selected every 200 kbp, and this dataset was used by Treemix version 1.13 [158] to fit migration edges on a bifurcating tree.

### *δaδi* demographic model

The demography model underlying the evolution of circum-basmati rice was tested using the diffusion approximation method of *δaδi* [96]. A visual representation of the 13 demographic models that were examined can be seen in Additional file 1: Figure S7. The population group and genotype calls used in the twisst analysis were also used to calculate the site allele frequencies. To conduct a *δaδi* analysis for three populations with polarized allele frequency spectrum, the polymorphic sites were polarized using the *O. barthii* reference genome. Using the Basmati 334 reference genome, the *O. barthii* genome was aligned using the same procedure outlined in the section “Whole-genome alignment of *Oryza* genomes assembled de novo*.*” This genome alignment was then used to determine the outgroup sequence status for every polymorphic site.

We optimized the model parameter estimates using the Nelder-Mead method and randomly perturbed the parameter values for four rounds. Parameter values were perturbed for threefold, twofold, twofold, and onefold in each subsequent round, while the perturbation was conducted for 10, 20, 30, and 40 replicates in each subsequent round. In each round, parameter values from the best likelihood model of the previous round were used as the starting parameter values for the next round. Parameter values from the round with the highest likelihood were chosen to parameterize each demographic model. Akaike Information Criteria (AIC) values were used to compare demography models. The demography model with the lowest AIC was chosen as the best-fitting model.

### Agronomic trait measurements

Data on geolocation of collection as well as on seed dimensions and seed weight for each of the circum-basmati landrace varieties included in this study were obtained from passport data included in the online platform Genesys (https://www.genesys-pgr.org/welcome).

## Supplementary information

**Additional file 1: Figure S1.** Dot plot comparing chromosome 6 of japonica variety Nipponbare to *circum*-aus variety N22 and indica variety R498. **Figure S2.** Distribution of the proportion of missing nucleotides for japonica variety Nipponbare gene models across the orthologous non-japonica genomic regions. **Figure S3.** Effect of coverage threshold to call a deletion and the total number of deletion calls for samples with various genome coverage. **Figure S4.** Density of presence-absence variation (PAV) per 500,000 bp window for each chromosome. **Figure S5.** Insertion time of LTR retrotransposon in various *Oryza* variety group genomes. **Figure S6.** Approximately Unbiased (AU) test result for a 4 population. **Figure S7.** Treemix result for japonica, *circum*-basmati, *circum*-aus, *O. rufipogon*, and outgroup *O. barthii*. **Figure S8**. Genome-wide topology weight from 500 SNP size window. **Figure S9.** 13 demographic models tested by a i. **Figure S10.** a i model fit for the best-fitting demographic model. **Figure S11.** Neighbor-joining phylogenetic tree of the 78 *circum*-basmati population sample. **Figure S12.** Proportion of ancestry plot for K = 2 to 5 across the 78 circum-basmati rice varieties, and the japonica and circum-aus population studied in Fig. 6c.

**Additional file 2: Table S1.** Inversion detect by *sniffles* in the Nipponbare reference genome. **Table S2.** The 78 *circum*-basmati samples with Illumina sequencing result used in this study. **Table S3.** Names of the Basmati 334 and Dom Sufid genome gene models that had a deletion frequency of zero across the population. **Table S4.** Names of the Basmati 334 and Dom Sufid genome gene models that had a deletion frequency of above 0.3 and omitted from down stream analysis. **Table S5.** Orthogroup status for the Basmati 334, Dom Sufid, R498, Nipponbare, and N22 genome gene models. **Table S6.** Count and repeat types of the presence-absence variation (PAV) in the Basmati 334 or Dom Sufid genome in comparison to the Nipponbare genome. **Table S7.** Gene ontology results for orthogroups where gene members from the *circum*-basmati are missing. **Table S8.** Gene ontology results for orthogroups where gene members from *circum*-aus, indica, and japonica are missing. **Table S9.** Population frequency across the 78 *circum*-basmati samples for orthogroups that were specifically missing a gene in the Basmati 334 and Dom Sufid genome gene models. **Table S10.** Genome coordinates of the LTR retrotransposons of the Basmati 334 genomes. **Table S11.** Genome coordinates of the LTR retrotransposons of the Dom Sufid genomes. **Table S12.** Genome coordinates of the Gypsy elements indicated with a single star in Fig. 3. **Table S13.** Genome coordinates of the Copia elements indicated with a single star in Fig. 3. **Table S14.** Genome coordinates of the Gypsy elements indicated with a double star in Fig. 3. **Table S15.** Genome coordinates of the Copia elements indicated with a triple star in Fig. 3. **Table S16.** The 82 *Oryza* population samples with Illumina sequencing result used in this study. **Table S17.** a i parameter estimates for the 13 different demographic models. See Additional file 1: Figure S9 for visualization of the estimating parameters.

**Additional file 3.** Review history.

## Data Availability

Raw nanopore sequencing FAST5 files generated from this study are available at the European Nucleotide Archive under bioproject ID PRJEB28274 (ERX3327648-ERX3327652) for Basmati 334 [159] and PRJEB32431 (ERX3334790-ERX3334793) for Dom Sufid [160]. Associated FASTQ files are available under ERX3498039-ERX3498043 for Basmati 334 and ERX3498024-ERX3498027 for Dom Sufid. Illumina sequencing generated from this study can be found under bioproject ID PRJNA422249 [161] and PRJNA557122 [162]. A genome browser for both genome assemblies can be found at http://purugganan-genomebrowser.bio.nyu.edu/cgi-bin/hgTracks?db=Basmati334 for Basmati 334 and http://purugganan-genomebrowser.bio.nyu.edu/cgi-bin/hgTracks?db=DomSufid for Dom Sufid. All data including the assembly, annotation, genome alignment, and population VCFs generated from this study can be found at Zenodo (10.5281/zenodo.3355330) [163].
