# Supplementary material for: Nanopore sequencing-based genome assembly and evolutionary genomics of circum-basmati rice
Source: Genome Biol. 2020 Feb 5;21:21. doi: 10.1186/s13059-020-1938-2 (PMC7001208; doi:10.1186/s13059-020-1938-2)
Supplement: Supplementary file 1 — Additional file 1: Figure S1. Dot plot comparing chromosome 6 of japonica variety Nipponbare to circum-aus variety N22 and indica variety R498. Figure S2. Distribution of the proportion of missing nucleotides for japonica variety Nipponbare gene models across the orthologous non-japonica genomic regions. Figure S3. Effect of coverage threshold to call a deletion and the total number of deletion calls for samples with various genome coverage. Figure S4. Density of presence-absence variation (PAV) per 500,000 bp window for each chromosome. Figure S5. Insertion time of LTR retrotransposon in various Oryza variety group genomes. Figure S6. Approximately Unbiased (AU) test result for a 4 population. Figure S7. Treemix result for japonica, circum-basmati, circum-aus, O. rufipogon, and outgroup O. barthii. Figure S8. Genome-wide topology weight from 500 SNP size window. Figure S9. 13 demographic models tested by a i. Figure S10. a i model fit for the best-fitting demographic model. Figure S11. Neighbor-joining phylogenetic tree of the 78 circum-basmati population sample. Figure S12. Proportion of ancestry plot for K = 2 to 5 across the 78 circum-basmati rice varieties, and the japonica and circum-aus population studied in Fig. 6c. [file 13059_2020_1938_MOESM1_ESM.docx]

**Supplementary Materials for:**

**Nanopore sequencing-based genome assembly and evolutionary genomics of basmati rice**

Jae Young Choi^1^, Zoe N. Lye^1^, Simon C. Groen^1^, Xiaoguang Dai^2^, Priyesh Rughani^2^, Sophie Zaaijer^3^, Eoghan D. Harrington^2^, Sissel Juul^2^ and Michael D. Purugganan^1,4^

^1^Center for Genomics and Systems Biology, Department of Biology, New York University, New York, New York, USA

^2^Oxford Nanopore Technologies, New York, New York, USA

^3^New York Genome Center, New York, New York, USA

^4^Center for Genomics and Systems Biology, NYU Abu Dhabi Research Institute, New York University Abu Dhabi, Abu Dhabi, United Arab Emirates

This file includes:

Figures S1 to S12

**Figure S1.** Dot plot comparing chromosome 6 of japonica variety Nipponbare to *circum*-aus variety N22 and indica variety R498.

**Figure S2.** Distribution of the proportion of missing nucleotides for japonica variety Nipponbare gene models across the orthologous non-japonica genomic regions.

**Figure S3.** Effect of coverage threshold to call a deletion and the total number of deletion calls for samples with various genome coverage.

**Figure S4.** Density of presence-absence variation (PAV) per 500,000 bp window for each chromosome.

**Figure S5. Insertion time of LTR retrotransposon in various *Oryza* variety group genomes.** Number of annotated LTR retrotransposons is shown above boxplot. The variety group genomes that do not have a significantly different insertion time after a Tukey’s range test are indicated with the same letter.

**Figure S6. Approximately Unbiased (AU) test result for a 4 population**. The total number and percentage of genes supporting the 15 possible topology involving the 4 population: japonica (J) Nipponbare, *circum*-basmati (B) Basmati 334 or Dom Sufid, *circum*-aus (A) N22, and *O. rufipogon* (R) after an Approximately Unbiased (AU) test. The top row numbers represent AU test results using Basmati 334 and bottom row represent AU test results using Dom Sufid.

**Figure S7. Treemix result for japonica, circum¬-basmati, circum-aus, O. rufipogon, and outgroup O. barthii.** (A) Treemix graphs from m=0 to 3. (B) Treemix log-likelihood of each migration model.

**Figure S8. Genome-wide topology weight from 500 SNP size window.** Chromosomal distribution of topology weights involving trios of the circum-aus, circum-basmati, and japonica rices (left), and the sum of the topology weights (right).

**Figure S9. 13 demographic models tested by aδi.**

**Figure S10. aδi model fit for the best-fitting demographic model.** Above row shows the observed and model fit folded site frequency spectrum. Below shows the map and histogram of the residuals.

**Figure S11. Neighbor-joining phylogenetic tree of the 78 *circum*-basmati population sample.**

**Figure S12. Proportion of ancestry plot for K = 2 to 5 across the 78 circum-basmati rice varities, and the japonica and circum-aus population studied in Figure 6C.** The color-coding from Figure 7A is indicated above each basmati sample’s ancestry proportion.
