# Supplementary material for: Nanopore sequencing-based genome assembly and evolutionary genomics of circum-basmati rice
Source: Genome Biol. 2020 Feb 5;21:21. doi: 10.1186/s13059-020-1938-2 (PMC7001208; doi:10.1186/s13059-020-1938-2)
Supplement: Supplementary file 3 — Additional file 3. Review history. [file 13059_2020_1938_MOESM3_ESM.docx]

Review History

**First round of review**

**Reviewer 1**

**Are you able to assess all statistics in the manuscript, including the appropriateness of statistical tests used?** Yes, and I have assessed the statistics in my report.

**Comments to author:**

This paper is globally interesting and well written.

It highlights the newly produced de novo genome sequences for two cultivars representing the basmati rice obtained with the Nanopore technology.

High quality sequences are described and compared to the Japonica reference sequence. Interesting features are revealed, such as specific repeat expansions, as well as examples of presence/absence variants over close to 20 Mb. While synteny conservation and genome collinearity are high, significant structural variation is documented on a particular chromosome (Ch 6).

Then the sequences are used for comparisons with other materials, first to reveal the genetic composition of the two new sequences, in terms of admixture, then to investigate the genetic diversity within this varietal group. This latter part is less convincing.

Comparing phylogenetic topologies among three varieties has strong limitations when the number of possible phyla is high (here cBasmati plus Japonica, Indica, cAus and Rufipogon). It is often not clear what is being demonstrated. There are surprising formulations, such as “each cultivar was monophyletic”, or “[(japonica, *circum*-basmati), *O. rufipogon*] was the true species topology” when the idea is to describe admixture. Some conclusions seem rapid. For example, the topology grouping Indica and cBasmati is far from negligible, with a frequency only 30% lower than the cAus with cBasmati topology. The mention of chromosomes 10 and 11 as outgroups for admixture patterns is followed later only for chromosome 10. Lastly it is not clear what the global evolutionary scenario analysis brings to the study.

Then the section on diversity among *circum-*Basmati rice varieties is run using a wealth of data (derived from the 3000 genomes plus a few additional) and diverse analytical approaches are used before concluding that there are three groups with geographical coherence. The evidence for the three groups is very weak, based essentially on two axes of a PCA, without clear confirmation by the fastSTRUCTURE or the “phylogenetic” tree. There is hardly any reference to the many studies that have addressed this topic previously. Notably there lack comparisons with the studies by Roy et al published in 2015 and Islam et al in 2018, and to two whole-genome studies by Civan et al (yet quoted in this study just for the cytoplasmic variation) and by Santos et al, both published earlier in 2019. Altogether the evidence shown suggests an East-West geographic cline with the fine-grained true basmati or sadri varieties in the West and coarser grains in the East, which is not new.

It is also questionable why a non-aromatic “Basmati” variety was chosen as a representation of the Basmati rice (title) while “basmati” etymologically means fragrant… Altogether the matter brought is actually limited and I am not convinced this reaches the standards for Genome Biology.

**Reviewer 2**

**Are you able to assess all statistics in the manuscript, including the appropriateness of statistical tests used?** Yes, and I have assessed the statistics in my report.

**Comments to author:**

The manuscript by Choi et al. untitled "Nanopore-based genome assembly and the evolutionary genomics of basmati rice" presents de novo assemblies of two rice landrace genomes from the basmati group, a group for which genomes were currently lacking (a single assembly is available for an elite line). The two landraces have distinct characteristics: Basmati 334 is a lowland Pakistanese landrace tolerant to drought and resistant to a broad spectrum of blight, while Dom Sufid is an Iranian variety susceptible to disease and drought but much appreciated for its aromatic taste and texture. The paper is really well framed and written. The methods are appropriate and conclusions reflect results. It is of interest and is of key importance for future work on rice. Its main focus is the genome assemblies, which are undoubtedly of good quality (given their length, N50, and coverage of BUSCO genes). Below I am suggesting some modifications that could help reinforcing the evolutionary analyses.

-     Some genes are present/absent. The authors describe those comparisons and established that basmati genes share the highest number of orthogroups with aus. It will be interesting to extend this comparison to more functional aspects. What are the genes unique to a genome are doing? Are they involved in some specific functions? What is the level of their expression (I believe they are expression data in rice that could be used to answer this question). Do they exhibit any specific pattern? I was not clear to me, which proportion of these genes were included in larger PAVs. Maybe the paragraphs and gene presence/absence and PAV description could be merged and shortened.

-     The authors mention a domestication gene Awn3-1. It would be nice to look at additional rice domestication genes in this basmati group and discuss the findings (maybe add a specific paragraph on domestication genes).

-     I would be interested on seeing a Treemix analysis with split and admixture results. I would include the wild relatives as well as the other divergent rice variety groups together. That would complement the ABBA-BABA test that is performed separately for japonica/basmati/rufipogon and aus/indica/basmati.

-     The authors have gathered population data from aus, basmati and japonica. I would really like to see a fast structure analysis that includes all of these accessions. Fast structure was ran only for the basmati group. It is possible that admixture from aus and indica varies from one genetic group to another.

-     Supl Figure 6: it would help to recall the topologies.

**Authors Response**

**Point-by-point responses to the reviewers’ comments:**

**Reviewer #1:** This paper is globally interesting and well written. It highlights the newly produced de novo genome sequences for two cultivars representing the basmati rice obtained with the Nanopore technology. High quality sequences are described and compared to the Japonica reference sequence. Interesting features are revealed, such as specific repeat expansions, as well as examples of presence/absence variants over close to 20 Mb. While synteny conservation and genome collinearity are high, significant structural variation is documented on a particular chromosome (Ch 6). Then the sequences are used for comparisons with other materials, first to reveal the genetic composition of the two new sequences, in terms of admixture, then to investigate the genetic diversity within this varietal group. This latter part is less convincing.

*Reply: We thank the reviewer for these suggestions. Our responses can be found below. Please view under Microsoft Word "Final Showing Markup" mode under the review tab so the page number and line number matches up.*

- Comparing phylogenetic topologies among three varieties has strong limitations when the number of possible phyla is high (here cBasmati plus Japonica, Indica, cAus and Rufipogon). It is often not clear what is being demonstrated. There are surprising formulations, such as "each cultivar was monophyletic", or "[(japonica, circumbasmati), O. rufipogon] was the true species topology" when the idea is to describe admixture.

*Reply: The reviewer’s point that our topological comparisons have limitations given the high number of possible phyla is a point well taken. However, it is because of the complications of inferring evolutionary relationships between a high number of phyla that we divided our species set into smaller samples, which made it possible to implement more rigorous phylogenetic and statistical testing. An illustrative example of this approach can be found in the human evolution literature, in which the evolutionary relationships between multiple human (sub-)populations are investigated. These investigations inevitably are confronted with the same complex problem of having to resolve relationships between a high number of “phyla”. The approach taken by many of these studies - the same approach we took - is to divide the many populations into specific trios or quartets, where members consist of populations of specific interest to the researcher (i.e. a trio of groups can be Africans, Europeans, and African Americans to investigate the evolutionary relationships between sub-populations as a result of the North America Slavery period; or the trio can consist of people of African, East Asian, and Native American ancestry to investigate the evolutionary history of the transSiberian crossing, and so on). A deeper methodological background of this approach, and the reasoning behind it, can be found in work by Patterson, Reich, and colleagues (https://www.ncbi.nlm.nih.gov/pmc/articles/PMC3522152/).*

*Following the same reasoning, our approach broke down a complex evolutionary relationship into its core components. The first main question we asked was what the relationship between japonica rice and basmati rice is; and following on from this question, we asked what the relationship of both of these groups is to the potential progenitor wild rice O. rufipogon. Given these questions, we conducted subsequent analysis using the trio japonica, basmati, and O. rufipogon. The second main question we asked is what the evolutionary relationship is between basmati and aus or indica rice, given that, although these two rices are evolutionarily most distant from basmati, there was the possibility of admixture occurring. Hence, by breaking up our sample set into smaller groups suited to our questions, we were able to conduct rigorous tests of our hypotheses on the specific evolutionary relationships between samples. This approach sets our analyses apart from previously published genome-wide analyses, since we used specific evolutionary tests such as the ABBA-BABA statistic, the Approximately Unbiased (AU) test of topology testing, and the dadi demographic analysis. We essentially conducted hypothesis tests of multiple rice evolutionary scenarios. While we acknowledge the limitations of our approach, we think that breaking down the high number of phyla into its core components, and subsequently testing specific evolutionary scenarios provides valuable new evidence for inferring the domestication history of basmati rice.*

*With regards to the comment on the relationship “[(japonica, circum-basmati), O. rufipogon]”, we conducted additional analysis (described in pg25 L529-548) to make sure that the topological relationship was true. Specifically, we further tested whether the admixture occurring between aus and basmati did not bias the topological relationship “[(japonica, circum-basmati), O. rufipogon]”. Our results confirm that the relationship “[(japonica, circum-basmati), O. rufipogon]” is indeed true.*

- Some conclusions seem rapid. For example, the topology grouping Indica and cBasmati is far from negligible, with a frequency only 30% lower than the cAus with cBasmati topology. The mention of chromosomes 10 and 11 as outgroups for admixture patterns is followed later only for chromosome 10. Lastly it is not clear what the global evolutionary scenario analysis brings to the study.

*Reply: The reviewer is correct that the topology grouping indica and basmati is 30% lower in frequency than the one grouping aus and basmati, but we took this as an initial piece of evidence that supports the admixture between aus and basmati rice. It is certainly possible there is some degree of gene flow between indica and basmati rice. However, the main point of our analysis was that the extent of admixture between aus and basmati was much higher than that between indica and basmati.*

*The reason why we only mentioned chromosome 10, and not also chromosome 11, later is because it was the only chromosome that was statistically significant as an outlier for both single-nucleotide and structural variation.*

*Our detailed reply to the “global evolutionary analysis purpose” can be found in the reviewer’s previous comment. To reiterate briefly, we conducted a genome-wide evolutionary analysis because a combined approach of evolutionary testing and modeling to determine the specific evolutionary scenario that led to the basmati rice had not been taken before.*

-Then the section on diversity among circum-Basmati rice varieties is run using a wealth of data (derived from the 3000 genomes plus a few additional) and diverse analytical approaches are used before concluding that there are three groups with geographical coherence. The evidence for the three groups is very weak, based essentially on two axes of a PCA, without clear confirmation by the fastSTRUCTURE or the "phylogenetic" tree.

*Reply: Based on the reviewer’s suggestion we conducted a STRUCTURE analysis, which included the aus and japonica populations: the two core populations that led to basmati rice. Results showed that when K=5, including the 2 aus and japonica population samples, the basmati rices divided into the three genetic groups that we designated initially (please see Supplemental Figure 12 and pg 30 L652-662 for results). Furthermore, and importantly, we wanted to make sure the genetic grouping had an additional basis and was not merely a simple population genetic grouping. Indeed, we found that certain agronomic traits were also segregating according to the genetic grouping (Figure 7D). Based on our initial evidence and these new results, we think our three geographically separated genetic groups are real.*

- There is hardly any reference to the many studies that have addressed this topic previously. Notably there lack comparisons with the studies by Roy et al published in 2015 and Islam et al in 2018, and to two whole-genome studies by Civan et al (yet quoted in this study just for the cytoplasmic variation) and by Santos et al, both published earlier in 2019. Altogether the evidence shown suggests an East-West geographic cline with the fine-grained true basmati or sadri varieties in the West and coarser grains in the East, which is not new.

*Reply: After careful consideration of the reviewer’s comment, we would like to point out that the two references (Roy et al. 2015 Plos One, and Islam et al. 2018 Scientific Reports) the reviewer suggested were studies that described aromatic rice in general, but not circum-basmati rice specifically. Aromaticity is not a pre-requisite of circumbasmati rice, and in fact many circum-basmati rice varieties do not actually have any fragrance at all (Kovach et al. 2009 PNAS). The circum-basmati rice population is a unique genetic group of rice varieties found in South and Southeast Asia that may or may not be aromatic. This has been detailed in our introduction in pg4 L79-83. When looking at the two references suggested by the reviewer (Roy et al., and Islam et al.), the majority of the aromatic rice varieties studied by Roy et al. were in fact either japonica or indica varieties that happened to be fragrant, while Islam et al. did not determine whether the aromatic rices they studied were indeed circum-basmati varieties or could be fragrant japonica or indica rices. Hence, we did not include references to these two studies, nor did we cite other studies that looked at “aromatic rices” in general, when these in fact investigated non-circum-basmati, but fragrant, rices.*

*With regard to Civan et al. 2019 GBE, we quoted the cytoplasmic variation from these authors, since the same group published their analyses on basmati rice evolution based on nuclear genomic data in a previous study (Civan et al. 2015 Nature Plants). We have appropriately cited both studies in pg32 L703-710. We have incorporated the reviewer’s other suggestion and have cited the Santos et al. 2019 GBE study in pg33 L713-716.*

*However, we respectfully disagree with the reviewer’s comment that the East-West geographic cline was already known. While we have not explicitly claimed novelty of our population relationship results, the citations the reviewer suggested do not already show the East-West cline. The studies by Roy et al. and Islam et al. described “aromatic rices”, where it was not certain if those rices were indeed true basmati rices (see our comments above). Furthermore, the other studies (Civan et al. and Santos et al.) have focused on the genome-wide japonica and aus admixture that led to the basmati rices, but not on the geography-based genetic structure of basmati rice. To our knowledge, this study is the first to investigate the genetic and geographic structure of basmati rice, and we further examined if these genetic groups had any phenotypic differences among them by testing if agronomic traits associated with the genetic structuring (See Fig 7C and D, for example). Thus, we think that our basmati rice population genomic results are novel.*

- It is also questionable why a non-aromatic "Basmati" variety was chosen as a representation of the Basmati rice (title) while "basmati" etymologically means fragrant.

*Reply: While the name “Basmati” has etymological roots to mean fragrance, not all basmati rices are fragrant, and not all fragrant rices are basmati varieties. In fact, several japonica or indica rices are also fragrant, indicating that fragrance is not a basmati rice-specific trait. This was described in our manuscript in pg 4 L78-83. In addition, unlike other de novo genome assembly projects in which breeding lines were sequenced, we have sequenced two traditional circum-basmati varieties (landraces) to capture the genomic variation that existed before modern agricultural practices. Hence, we would argue that our circum-basmati rices represent the most “basmati-like” genetic variations and were valid choices for genome sequencing. Lastly, the Dom Sufid variety we sequenced is in fact a fragrant variety (see Kovach et al. 2009 PNAS for the fragrance test for Dom Sufid, and pg 12 L227-241 of this manuscript where we examined the fragrance gene location that confirms Dom Sufid carries the causal mutation for fragrance). This should address the reviewer’s concern, given that with our choice of varieties we have captured the genetic and phenotypic variation for fragrance that exists within the circum-basmati rices.*

**Reviewer #2:** The manuscript by Choi et al. untitled "Nanopore-based genome assembly and the evolutionary genomics of basmati rice" presents de novo assemblies of two rice landrace genomes from the basmati group, a group for which genomes were currently lacking (a single assembly is available for an elite line). The two landraces have distinct characteristics: Basmati 334 is a lowland Pakistanese landrace tolerant to drought and resistant to a broad spectrum of blight, while Dom Sufid is an Iranian variety susceptible to disease and drought but much appreciated for its aromatic taste and texture. The paper is really well framed and written. The methods are appropriate and conclusions reflect results. It is of interest and is of key importance for future work on rice. Its main focus is the genome assemblies, which are undoubtedly of good quality (given their length, N50, and coverage of BUSCO genes). Below I am suggesting some modifications that could help reinforcing the evolutionary analyses.

*Reply: We thank the reviewer for the encouragement and feedback. Below we have addressed the comments made by the reviewer. Please view under Microsoft Word "Final Showing Markup" mode under the review tab so the page number and line number matches up.*

- Some genes are present/absent. The authors describe those comparisons and established that basmati genes share the highest number of orthogroups with aus. It will be interesting to extend this comparison to more functional aspects. What are the genes unique to a genome are doing? Are they involved in some specific functions? What is the level of their expression (I believe they are expression data in rice that could be used to answer this question). Do they exhibit any specific pattern? I was not clear to me, which proportion of these genes were included in larger PAVs. Maybe the paragraphs and gene presence/absence and PAV description could be merged and shortened.

*Reply: We divided the presence/absence variation (PAV) analysis into two major parts: 1) the genome-wide PAV (from pg 15 L278-319), and 2) the gene-specific PAV across the basmati genome sequences (from pg 17 L321-375). For the gene-specific PAV analysis we edited the title (pg 17 L321) to clearly indicate the analysis was different from the previous section’s genome-wide PAV analysis. Further, in the gene-specific PAV analysis section in pg 17 L321-329 and Supplemental Table 7 and 8, we conducted gene ontology analysis to determine the gene functions that were enriched among basmati rice-specific gene deletions and retentions.*

*As mentioned by the reviewer, ultimately, this was done to infer the potential functional effects from these gene gains and losses, in particular given that we did not have gene expression data for these basmati rices. While we agree with the reviewer’s comment that gene expression data would give greater insight, we would have needed expression data specifically from our basmati varieties. In addition, expression data from multiple tissues or conditions would have been necessary to infer the function of these genic PAVs. We think this would be suitable for a potential follow up study. Given that most genome-wide structural variation analysis in rice has focused on the genic space, we wanted to report on the variation that exists in the non-genic space as well. Hence, we would like to keep the two parts (i.e. the non-genic vs. genic space PAV analysis) separate. We hope this distinction now makes the results section clearer.*

- The authors mention a domestication gene Awn3-1. It would be nice to look at additional rice domestication genes in this basmati group and discuss the findings (maybe add a specific paragraph on domestication genes).

*Reply: We have incorporated the reviewer’s suggestion and examined the gene deletion status for core rice domestication genes in the basmati population (we have noted this result in pg19 L362-375). We observed that many of the genes that were involved in the initial domestication phase of Asian rice were not deleted in the basmati population. We think this is because basmati rice has a domestication history that is only partially shared with the other major Asian rice subpopulations. In many of the domestication-associated genes the causal domestication mutation is not a gene deletion. Indeed, we think many of the gene deletions that were selected in basmati rice occurred during the cultivation phase of basmati domestication, when basmati ricespecific mutations were selected.*

- I would be interested on seeing a Treemix analysis with split and admixture results. I would include the wild relatives as well as the other divergent rice variety groups together. That would complement the ABBA-BABA test that is performed separately for japonica/basmati/rufipogon and aus/indica/basmati.

*Reply: We have incorporated the reviewer’s suggestion and have conducted a treemix analysis with the populations basmati, aus, japonica, O. rufipogon, and O. barthii. The issue, however, with the wild rices is that a previous study by Wang et al. 2017 Genome Res has reported that Asian wild rices have recently admixed with domesticated rice, and that many of these wild individuals likely represent feralized rice populations. Hence, we analyzed an O. rufipogon dataset from a different study (Xu et al. 2012 Nat Biotech), which had not been subject of evolutionary analyses (see pg 45 L984-991 for details on sampled population and methodology). However, our treemix analysis still detected evidence of admixture occurring between the O. rufipogon population and the basmati rice population. We think it is currently difficult (if not impossible) to study the evolutionary relationship between wild and domesticated rice, since the recent admixture between the two groups has essentially wiped out the historical progenitor-domesticate evolutionary signatures. We do, however, still detect the evidence of admixture between aus and basmati. Our results can be found in pg 27 L579-588 of the manuscript.*

- The authors have gathered population data from aus, basmati and japonica. I would really like to see a fast structure analysis that includes all of these accessions. Fast structure was ran only for the basmati group. It is possible that admixture from aus and indica varies from one genetic group to another.

*Reply: We have incorporated the reviewer’s suggestion and conducted a STRUCTURE analysis, which included our basmati population and the japonica/aus populations (results can be found in Supplementary Figure 12). We focused on the japonica and aus populations, because we showed that these are the two most important rice subpopulations that contributed genetic material to basmati rice. When we examined the results in the way the reviewer has suggested, we indeed observed 3 different basmati genetic groups. The groups were divided based on the amount of japonica or aus ancestry each of them contained. The results are noted in pg30 L652- 662.*

- Supl Figure 6: it would help to recall the topologies.

*Reply: We have added in the topologies for Supplementary Fig 8 (which was Supplemental Fig 6 in the previous manuscript version) for clarity.*

**Second round of review**

**Reviewer 1**

Altogether I find the new version better than the first one and the response of the authors is generally acceptable and enlightening.

Yet I shall comment two topics.

1. On the title.
I mentioned that basmati literally means fragrant. Two variety genomes were sequenced. The first variety (not aromatic) is not a true basmati despite its name and the second is a sadri variety. I agree that the two genomes altogether can be used to represent the circum-Basmati group, yet I am not at ease with the title that mentions the “evolutionary genomics of basmati rice”

2. On the specific topic of the novelty of the results on variation within the cBasmati group.
It seems to me that the general features that are brought about by this study were given as early as the initial large scale description of the species based on isozymes and further studies with SSRs.
F

rom Glaszmann (Theor Appl Genet 1987, quoted by the authors), one can read : “Group V spreads from Iran to Burma. It consists of very diverse varieties, many of which are considered as high quality rices, such as the Sadri rices from Iran, the Basmati rices from Pakistan, India and Nepal and some rices from Burma which also have a very high cooking elongation”.

And from Glaszmann (Genome 1988): “Within group V, the geographic pattern of  variation (Table 8) was complex. It appeared to be longitudinally structured at locus Amp-3. The varieties from Iran, which are geographically separated from the other Asian varieties, showed particular traits, especially at loci Pgi-2 and Cat-1”.

Then a study focused on aromatic rices from Myanmar using SSR markers (Myint et al , Rice 2012) highlighted (in its abstract)  that: “They revealed a new cluster of accessions close to, but distinct from, non-Myanmar varieties in group 5. With reference to earlier terminology, we propose to distinguish a group “5A” including group 5 varieties from the Indian subcontinent (South and West Asia) and a group “5B” including most group 5 varieties from Myanmar …. Other accessions, all of group 5B, displayed a particular BADH2 allele with a 3 bp insertion and 100% association with aroma”.

Thus before the study presented here, the literature mentioned a longitudinal structure between Iran and Myanmar, with accessions from Iran showing specific alleles at several key isozyme loci and accessions from Myanmar exhibiting specific SSR profiles as well as a BADH2 allele. This starting point has been overlooked in the manuscript.

While the current study is based on immensely many more polymorphisms, it rests on fewer accessions, which makes it difficult to address geographic structure. The results seem to me compatible and corroborative with earlier characterizations but the sample size is too low to conclude a structure into three groups; there are above 10% intermediates, concordance is incomplete between the origins and the groups (some countries appear in several groups, India in all three groups) and one group is qualified as “paraphyletic” (line 631, figure 11). The proposed classification will probably have to be modified in the future when more accessions are studied and this would thus be a source of confusion.

While this does not affect the global value of the information generated and described here, reference to earlier work and strength of the conclusions on the subgrouping should be adjusted, I think.

**Reviewer 2**

I have read the revised version of the manuscript. I am satisfied with the changes they made. The authors carefully considered all my recommendations and clarified the issues that I have raised in my first review and amended their text accordingly.

**Authors Response**

**Point-by-point responses to the reviewers’ comments:**

*REPLY: To accommodate the reviewer 1’s first comment we have edited the title from “Nanopore sequencing-based genome assembly and evolutionary genomics of basmati rice” to “Nanopore sequencing-based genome assembly and evolutionary genomics of circum-basmati rice”.*

*In addition, to address reviewer1’s second comment we have inserted the following paragraph in the discussion section where we discuss the interpretation of the genome-wide population structure results (it was the last paragraph of the discussion). Note that the below paragraph contains the three citations the reviewer suggested to include.*

*"Our genome-wide evidence of geographic structuring is also consistent with past studies that examined isozyme loci or simple sequence repeat (SSR) markers in Asian rice, and finding a longitudinal genetic structure from Iran to Myanmar. Isozymes could differentiate the circum-basmati rice from other Asian rice subpopulations (classified as group V in Glaszmann 1987) and the varities from Iran were especially differentiated at two isozyme loci (Glaszmann 1988). SSR markers differentiated the Myanmar group V varities from the rest of south and west Asian varities (Myint et al. 2012 Rice). Based on this study and past evidences, genome-wide survey of an expanded sample of circum-basmati should assist in determining the fine-scale genetic structure of the circum-basmati population."*
